# Therapeutic potential of berberine in attenuating cholestatic liver injury: insights from a PSC mouse model

**DOI:** 10.1186/s13578-024-01195-8

**Published:** 2024-01-25

**Authors:** Yanyan Wang, Derrick Zhao, Lianyong Su, Yun-Ling Tai, Grayson W. Way, Jing Zeng, Qianhua Yan, Ying Xu, Xuan Wang, Emily C. Gurley, Xi-Qiao Zhou, Jinze Liu, Jinpeng Liu, Weidong Chen, Phillip B. Hylemon, Huiping Zhou

**Affiliations:** 1 Department of Microbiology and Immunology, Virginia Commonwealth University and Richmond Veterans Affairs Medical Center, 1220 East Broad Street, MMRB-5044, Richmond, VA 23298-0678 USA; 2 School of Pharmaceutical Science, Anhui University of Chinese Medicine, Hefei, Anhui China; 3 Department of Endocrinology, Jiangsu Province Hospital of Chinese Medicine, Nanjing University of Chinese Medicine, Nanjing, China; 4 Department of Biostatistics, Virginia Commonwealth University, Richmond, VA USA; 5 Department of Computer Science, University of Kentucky, Lexington, KY USA

**Keywords:** Bile acids, Cholestasis, Inflammation, Berberine, Gut microbiome

## Abstract

**Background and aims:**

Primary sclerosing cholangitis (PSC) is a chronic liver disease characterized by progressive biliary inflammation and bile duct injury. Berberine (BBR) is a bioactive isoquinoline alkaloid found in various herbs and has multiple beneficial effects on metabolic and inflammatory diseases, including liver diseases. This study aimed to examine the therapeutic effect of BBR on cholestatic liver injury in a PSC mouse model (Mdr2^−/−^ mice) and elucidate the underlying mechanisms.

**Methods:**

Mdr2^−/−^mice (12–14 weeks old, both sexes) received either BBR (50 mg/kg) or control solution daily for eight weeks via oral gavage. Histological and serum biochemical analyses were used to assess fibrotic liver injury severity. Total RNAseq and pathway analyses were used to identify the potential signaling pathways modulated by BBR in the liver. The expression levels of key genes involved in regulating hepatic fibrosis, bile duct proliferation, inflammation, and bile acid metabolism were validated by qRT-PCR or Western blot analysis. The bile acid composition and levels in the serum, liver, small intestine, and feces and tissue distribution of BBR were measured by LC–MS/MS. Intestinal inflammation and injury were assessed by gene expression profiling and histological analysis. The impact on the gut microbiome was assessed using 16S rRNA gene sequencing.

**Results:**

BBR treatment significantly ameliorated cholestatic liver injury, evidenced by decreased serum levels of AST, ALT, and ALP, and reduced bile duct proliferation and hepatic fibrosis, as shown by H&E, Picro-Sirius Red, and CK19 IHC staining. RNAseq and qRT-PCR analyses indicated a substantial inhibition of fibrotic and inflammatory gene expression. BBR also mitigated ER stress by downregulating Chop, Atf4 and Xbp-1 expression. In addition, BBR modulated bile acid metabolism by altering key gene expressions in the liver and small intestine, resulting in restored bile acid homeostasis characterized by reduced total bile acids in serum, liver, and small intestine and increased fecal excretion. Furthermore, BBR significantly improved intestinal barrier function and reduced bacterial translocation by modulating the gut microbiota.

**Conclusion:**

BBR effectively attenuates cholestatic liver injury, suggesting its potential as a therapeutic agent for PSC and other cholestatic liver diseases.

**Supplementary Information:**

The online version contains supplementary material available at 10.1186/s13578-024-01195-8.

## Background

Primary sclerosing cholangitis (PSC) is a chronic cholestatic liver disorder characterized by inflammation and bile duct narrowing, which results in the accumulation of bile acids (BAs) in the liver, leading to hepatic damage, progressive liver fibrosis, cirrhosis, and even liver cancer [1, 2]. Over the last several decades, extensive efforts have been made to identify the mechanisms underlying cholestatic liver injury [3–5]. However, no effective therapy has been developed due to the complexity of disease pathogenesis. Liver transplantation remains the only life-extending treatment for end-stage PSC patients [4]. It has been well-accepted that dysregulation of BA homeostasis and inflammation are the major driving forces of the disease progression of PSC [6]. In addition, dysbiosis and intestinal barrier dysfunction also have been reported as key contributors to cholestatic liver injury [7–9]. Therefore, an effective therapeutic agent for PSC must be able to modulate bile acid metabolism, inflammatory response, and the gut microbiome.

Berberine (BBR), an isoquinoline alkaloid isolated from the rhizome of the herb *Coptis chinensis* and *Berberis vulgaris*, is one of the most commonly used plant medicines in China and Asia with various biological activities [10–13]. BBR has been reported as a promising therapeutic agent for cardiovascular and metabolic diseases, including metabolic dysfunction associated fatty liver disease (MAFLD) and diabetes [10, 14–17]. BBR’s beneficial effect on hepatic lipid homeostasis is achieved via regulating BA synthesis and excretion [15]. We have previously reported that BBR can inhibit HIV protease inhibitor-induced ER stress in macrophages and inhibit free fatty acid and LPS-induced inflammation via modulating ER stress response in macrophages and hepatocytes [17, 18]. Our recent study further showed that BBR could not only inhibit hepatic fibrosis by modulating the expression of multiple genes involved in hepatic stellate cell activation and cholangiocyte proliferation but also restore the BA homeostasis in the diet-induced metabolic dysfunction-associated steatohepatitis (MASH) mouse model [16]. However, the therapeutic effect of BBR on cholestatic liver disease has not been studied and is the primary focus of this study.

The current study tested the therapeutic effect of BBR on the cholestatic liver disease and further examined the potential cellular/molecular mechanisms using the best available PSC mouse model, Mdr2^−/−^ mice [19–21]. The results indicated that BBR significantly reduced cholestatic liver injury via modulating BA metabolism and gut microbiota, inhibiting the inflammatory response and ER stress and protecting intestinal barrier function.

## Results

### BBR improves cholestatic liver injury in Mdr2^−/−^ mice

**Fig. 1:**
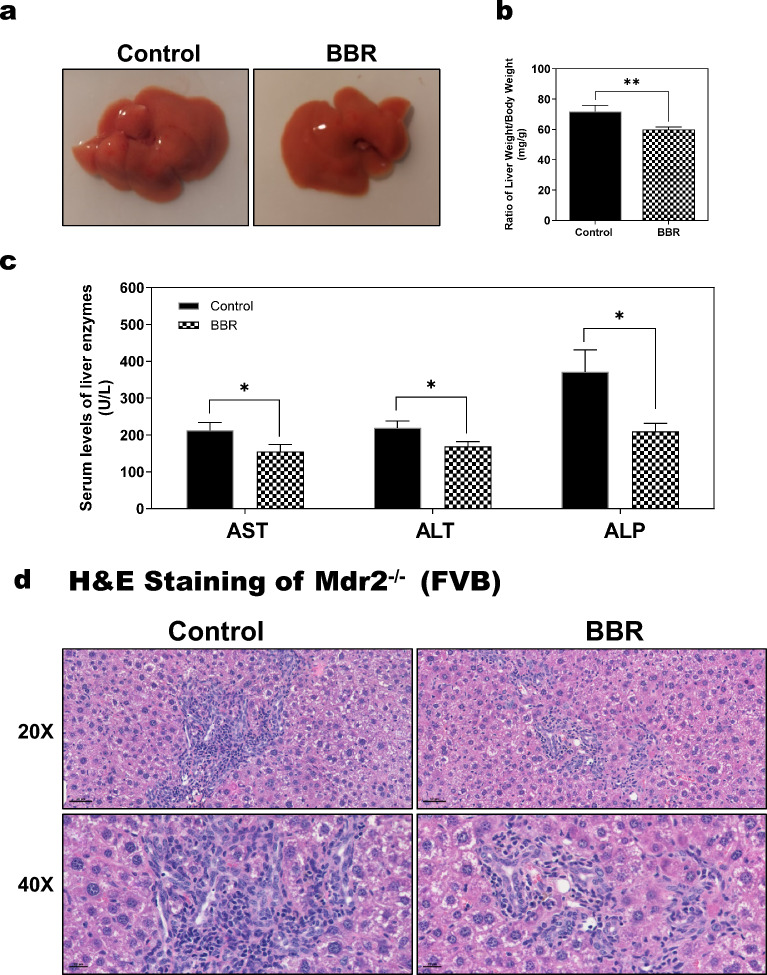
Effect of BBR on cholestatic liver injury in Mdr2^−/−^ mice. Mdr2^−/−^ mice (FVB background, both sexes) were treated with vehicle (Control group) or BBR (50 mg/kg) (BBR group) via oral gavage daily for 8 weeks. **a** Representative liver images. **b** The ratio of liver weight to body weight. **c** Serum liver enzyme levels (AST, ALT, ALP). **d** Representative images of the liver sections stained with H&E staining (scale bar, 50 µm for 20 × , 20 µm for 40 × magnification). Data are expressed as the mean ± SEM. Statistical significance relative to control: *p < 0.05, **p < 0.01 (n = 9–12)

Mdr2^−/−^ mice are a well-established model of cholestatic cholangiopathies [19–21]. To examine the therapeutic effect of BBR on cholestatic liver injury, Mdr2^−/−^ mice (FVB background, 12–14 weeks old) were treated with BBR (50 mg/kg) or vehicle by intragastric administration once daily for 8 weeks. As shown in Figs. 1a-b, BBR significantly reduced the ratio of liver weight to body weight compared to the control Mdr2^−/−^ mice. The serum alkaline phosphatase (ALP), aspartate aminotransferase (AST) and alanine aminotransferase (ALT) levels were also significantly reduced by BBR in Mdr2^−/−^ mice (Fig. 1c). The body weight and total serum albumin (ALB) levels remained unchanged (Additional file 1: Fig. S1a, b). Histological analysis showed that BBR treatment significantly reduced cholestatic liver injury as illustrated by H&E staining (Fig. 1d). To further verify the therapeutic effect of BBR on cholestatic liver injury, we did the same study using Mdr2^−/−^ mice with C57BL/6 background. As shown in Additional file 1: Fig. S1c, d, similar results were obtained. BBR treatment not only reduced the serum levels of AST, ALT, and ALP, but also reduced cholestatic liver injury as indicated by H&E staining.

### BBR reduces cholestatic liver injury by modulating global transcriptomic profile in Mdr2^−/−^ mice

**Fig. 2:**
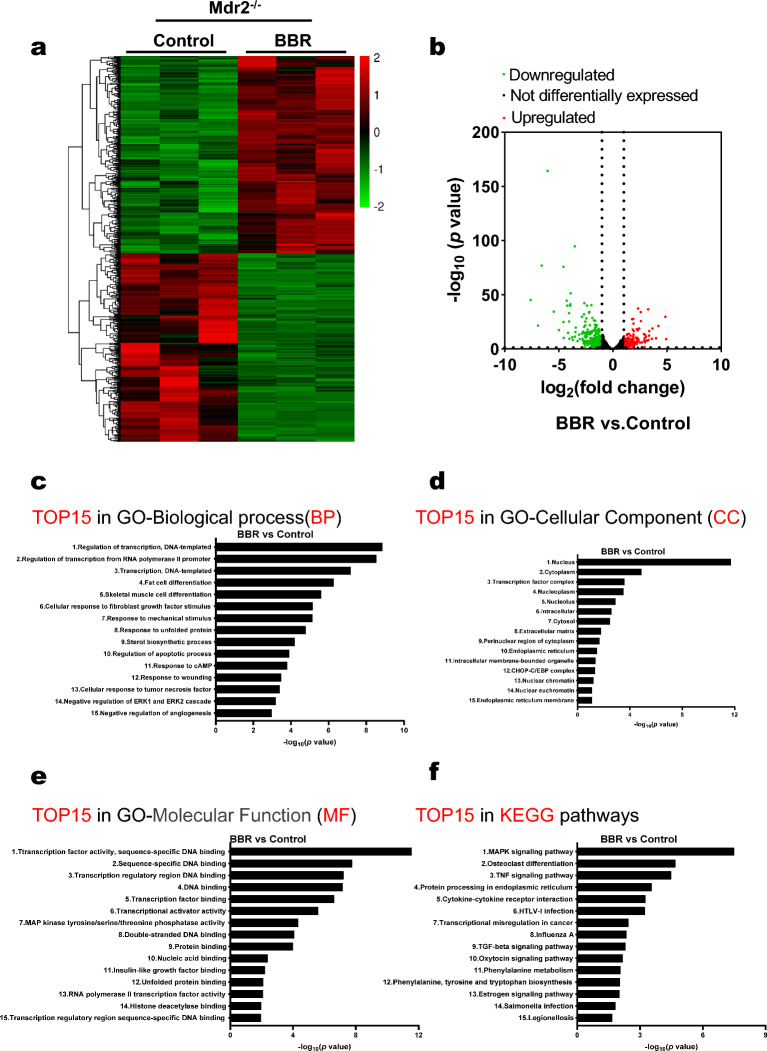
Transcriptomic Profiling and Pathway Analysis of Differentially Expressed Genes (DEGs) in BBR-treated Mdr2^−/−^ mice. Liver RNA samples from each experimental group (three per group) underwent total transcriptome sequencing (RNA-seq). DEGs between the BBR-treated and control groups were identified using fold change (FC) and p-values (FC ≥ 2 and p-value < 0.05). Subsequent Gene Ontology and Kyoto Encyclopedia of Genes and Genomes (KEGG) pathway analyses were conducted. **a** Hierarchical clustering heatmaps display DEGs, with a Z-score normalization of RNA-seq data. Red and green colors signify up- and down-regulated gene expression, respectively. **b** Volcano plots show gene expression differences; red dots represent upregulated genes, green dots for downregulated genes, and black dots for genes not differentially expressed. **c** Top 15 enriched biological processes (GO-BP), **d** cellular components (GO-CC), and, **e** molecular functions (GO-MF). **f** Highlights the top 15 enriched pathways according to KEGG analysis

To examine the underlying mechanisms by which BBR reduces cholestatic liver injury in Mdr2^−/−^ mice, total RNA transcriptome analysis was performed. As shown in Additional file 1: Fig. S2a, the heatmap showed distinct expression profiles between the WT and Mdr2^−/−^ mice, while the BBR treatment group showed a similar profile to the WT mice. The volcano plot further showed the change of gene expression induced in Mdr2^−/−^ mice was significantly reversed by BBR treatment (Figs. 2b and Additional file 1: Fig. S2b). As shown in Additional file 1: Fig. S2c, compared to the WT mice, the Mdr2^−/−^ mice exhibited an upregulation of 1260 genes and down-regulation of 677 genes, while BBR treatment down-regulated 287 genes and upregulated 300 genes, compared to vehicle-treated Mdr2^−/−^ mice. Furthermore, Gene Ontology analysis and Kyoto Encyclopedia of Genes and Genomes (KEGG) pathways analysis showed that BBR was able to impact the pathways in biological process, cellular components, and molecular function, such as regulation of transcription, cellular response to fibroblast growth factor stimulus, extracellular matrix, endoplasmic reticulum, etc. (Figs. 2c–f). IPA (Ingenuity Pathway Analysis) further showed that the hepatic fibrosis signaling pathway, tumor microenvironment pathway, IL-17 signaling, HER-2 signaling and GHRH signaling were activated in Mdr2^−/−^ mice, which were inhibited after BBR treatment (Additional file 1: Fig. S3). In addition, the activated senescence pathway was also inhibited by BBR.

### BBR attenuates bile duct injury and hepatic fibrosis in Mdr2^−/−^ mice

**Fig. 3:**
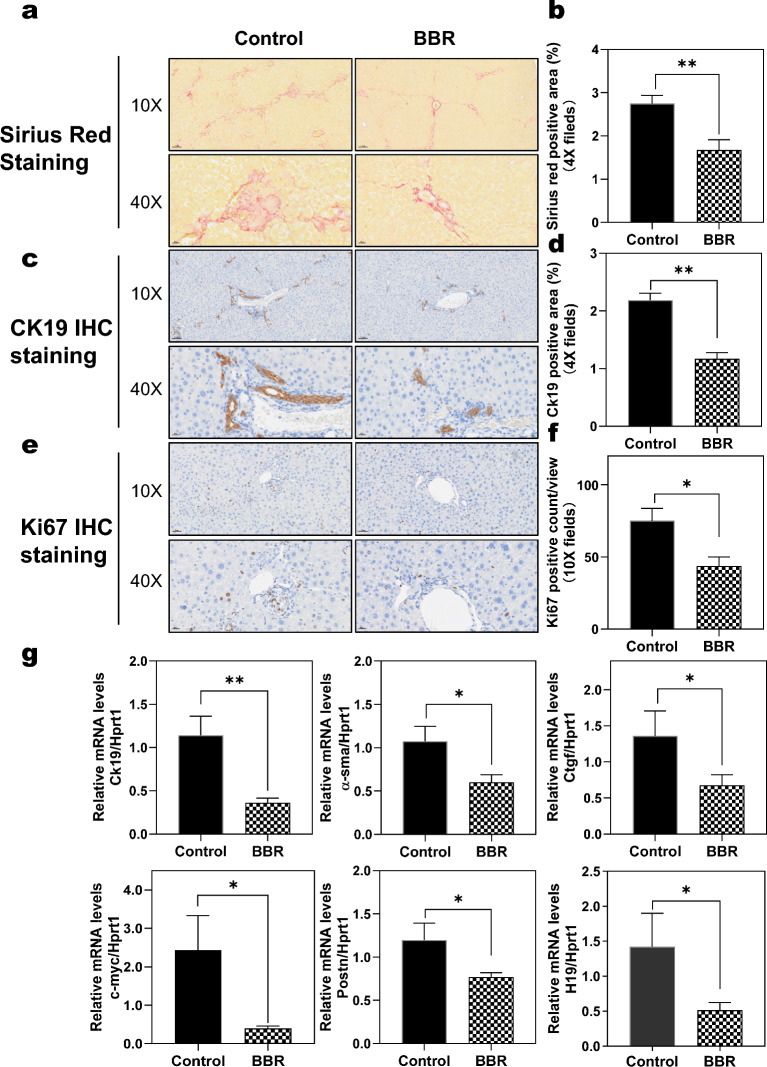
Effect of BBR on ductular proliferation and cholestatic liver fibrosis in Mdr2^−/−^ mice. **a** Representative images of liver sections stained with Picro-Sirius Red (scale bar, 100 µm for 10 × and 20 µm for 40 × magnification). **b** Quantification of Sirius red staining. **c** Representative images of IHC staining of CK19 (cytokeratin 19). **d** Quantified positive area of CK19 staining. **e** Representative images of IHC staining of Ki67. **f** Quantification of Ki67 positive cells. **g** Relative mRNA levels of key genes involved in ductular proliferation and cholestatic liver fibrosis: Ck19, α-Sma, Ctgf, C-myc, Postn, and H19. The mRNA levels were determined by real-time RT-PCR and normalized with HPRT1 as an internal control. Data are expressed as the mean ± SEM. Statistical significance relative to Control group is indicated as *p < 0.05, **p < 0.01 (n = 9–12)

The proliferation of bile ducts and hepatic fibrosis are associated with the development of cholestatic liver injury [22]. Picro-Sirius Red staining of the liver tissue sections showed that BBR treatment significantly reduced hepatic fibrosis in Mdr2^−/−^ mice, as shown in Figs. 3a-b. Importantly, BBR treatment significantly decreased proliferation of bile ducts as determined by IHC staining of keratin 19 (Krt19, also known as Ck19) and Ki67 (Figs. 3c-f and Additional file 1: Fig. S4a). In addition, BBR treatment significantly decreased the content of hepatic hydroxyproline in Mdr2^−/−^ mice (Additional file 1: Fig. S4b). As shown in the Fig. 3g and Additional file 1: Fig. S5a, major fibrotic genes were significantly downregulated after BBR treatment in Mdr2^−/−^ mice, such as Ck19, α-Sma (smooth muscle actin), Ctgf (connective tissue growth factor), C-myc (cellular myelocytomatosis oncogene), Postn (periostin, osteoblast specific factor), etc. Although RNA-seq data did not identify significant changes in H19 (long no coding RNA H19), the real-time RT-PCR analysis showed that BBR significantly reduced H19 expression. Moreover, BBR was able to reduce the mRNA levels of Pai1, Col12a1, Sox9, Egr1, Egr2, Egr3, Hbegf, Cyr61, and P4ha1 in Mdr2^−/−^ mice (Additional file 1: Fig. S5b).

### BBR reduces hepatic inflammation and stress in Mdr2^−/−^ mice

**Fig. 4:**
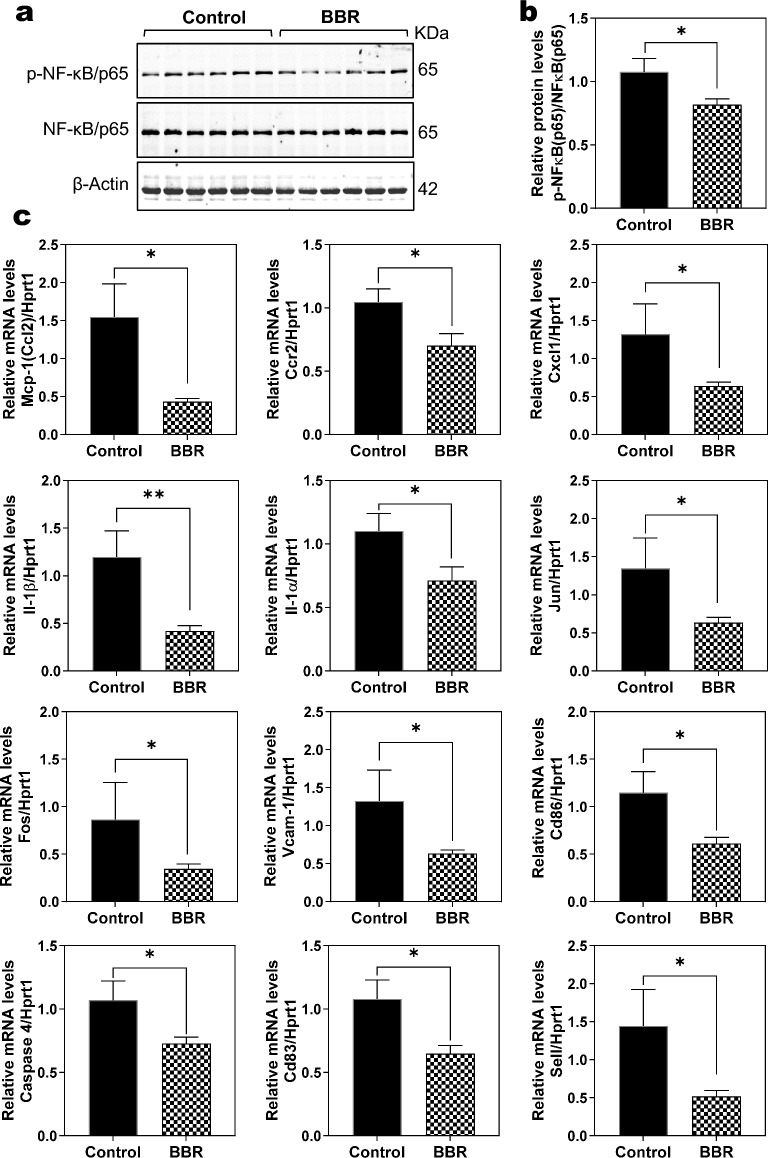
Effect of BBR on hepatic inflammation in Mdr2^−/−^ mice. **a** Representative immunoblot images of phosphorylated (p)-nuclear factor (NF)-κB/p65, total NF-κB/p65, and loading control β-Actin are shown. **b** The relative protein level of p-NF-κB/p65, normalized to total NF-κB/p65. **c** Relative mRNA levels of inflammation-related genes implicated in cholestatic liver injury, including Mcp-1(Ccl2), Ccr2, Cxcl1, IL-1α, IL-1β, Jun, Fos, Vcam-1, Cd86, Casp 4, Cd83 and Sell, normalized with HPRT1 as an internal control. Data are expressed as the mean ± SEM. Statistical significance relative to Control group: *p < 0.05, **p < 0.01 (n = 9–12)

Inflammation and oxidative stress response are key factors in cholestatic liver injury disease progression. The nuclear factor-kB (NF-κB) pathway is one of the main signaling pathways associated with inflammatory responses and plays a significant role in cholestatic liver disease progression [23]. As shown in Fig. 4a, b, Western blot analysis indicated that BBR treatment significantly reduced the activation of NF-κB in Mdr2^−/−^ mice. The RNA-seq data also showed that BBR significantly inhibited the expression of key genes involved in hepatic inflammation and stress (Additional file 1: Fig. S6). We further confirmed the expression levels of chemokine (C–C motif) ligand 2 (Ccl2/Mcp-1), chemokine (C–C motif) receptor 2 (Ccr2), chemokine (C-X-C motif) ligand 1 (Cxcl1), interleukin (IL)-1α, and IL-1β, jun proto-oncogene (Jun), FBJ osteosarcoma oncogene (Fos), vascular cell adhesion molecule 1 (Vcam-1), Cd86, caspase 4, and Cd83, selectin (Sell) using the real-time RT-PCR. As shown in Fig. 4c, BBR treatment significantly reduced the expression of these inflammatory genes in Mdr2^−/−^ mice. In addition, as shown in Additional file 1: Figs. S7–S9, pathway analysis showed that BBR was able to inhibit the activation of NF-kB signaling pathway, MAPK signaling pathway, and oxidative phosphorylation in Mdr2^−/−^ mice.

### BBR modulates endoplasmic reticulum (ER) stress in Mdr2^−/−^ mice

**Fig. 5:**
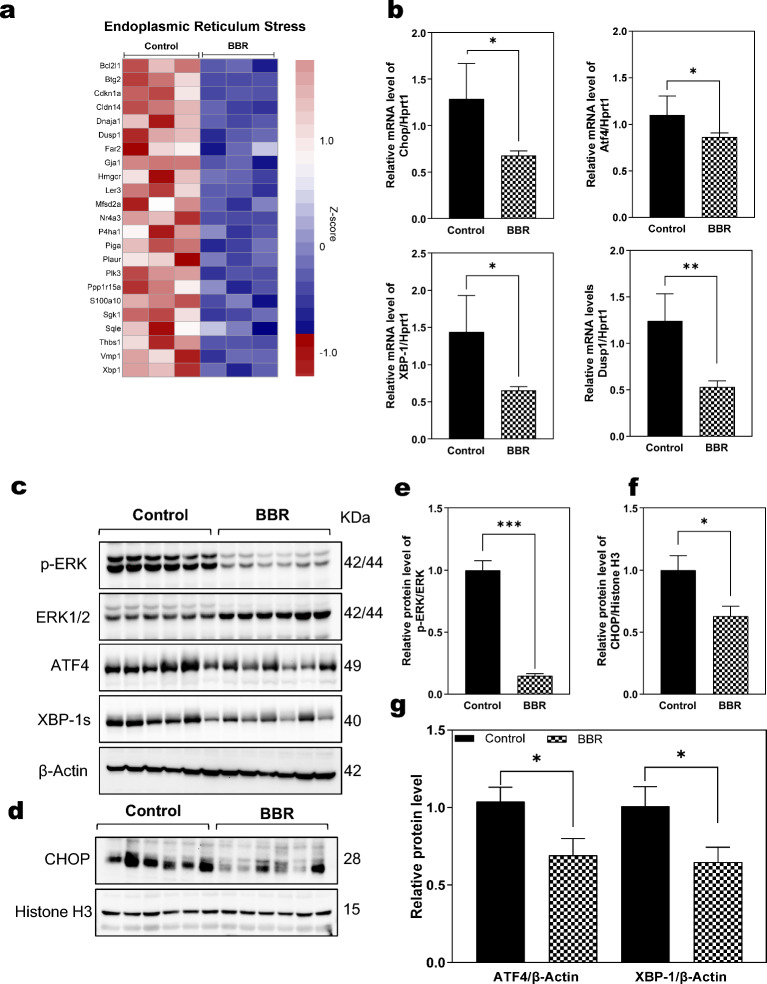
Effect of BBR on hepatic endoplasmic reticulum (ER) stress in Mdr2^−/−^ mice.** a** Representative heatmap of the key genes involved in ER stress response in the liver, comparing BBR-treated group with the Control group. The RNA-seq data were normalized using a Z-score for tag counts, with red and blue colors denoting high and low gene expression, respectively. **b** Relative mRNA expression levels of ER stress-related genes (Chop, Atf4, Xbp-1, and Dusp1), normalized with HPRT1 as an internal control. **c** Representative immunoblot images of phosphorylated (p)-ERK, total ERK, ATF4, and XBP-1. **d** Representative immunoblot images of nuclear CHOP. **e** Relative protein level of p-ERK, normalized using ERK as a loading control. **f** Relative protein level of CHOP, normalized with Histone H3 as a loading control. **g** Relative protein levels of ATF4 and XBP-1, normalized against β-actin as a loading control. Data are expressed as mean ± SEM. Statistical significance compared to the control group is indicated as *p < 0.05, **p < 0.01, ***p < 0.001 (n = 9–12)

Endoplasmic reticulum (ER) stress occurs when ER homeostasis is perturbed by the accumulation of unfolded/misfolded protein or calcium depletion [24]. Our previous studies and studies from others demonstrate a crucial role of ER stress in the development of liver fibrosis in cholestatic liver disease [7, 25]. As shown in Fig. 5a, the heatmap displayed a dramatic down-regulation of major genes in ER stress in Mdr2^−/−^ mice treated with BBR. To further confirm the findings of RNA-seq analysis, we measured both mRNA and protein expression levels of key genes involved in ER stress. As shown in Fig. 5b, consistent with RNA-seq analysis, real-time RT-PCR showed that BBR significantly downregulated the mRNA expression level of CCAAT/enhancer-binding protein homologous protein (Chop), activating transcription factor 4 (Atf4), X-box binding protein 1 (Xbp-1), and dual specificity phosphatase 1 (Dusp1) in Mdr2^−/−^ mice. Similarly, the Western-blot analysis further showed that BBR decreased the protein expression levels of p-ERK/ERK, ATF4, and XBP-1s in Mdr2^−/−^ mice (Figs. 5c, e, and g). As shown in figs. 5d, f, the nuclear protein expression of CHOP was significantly reduced by BBR treatment in Mdr2^−/−^ mice. Furthermore, as shown in Additional file 1: Fig. S10, pathway analysis showed that BBR was able to restore the dysregulation of protein processing in the ER in Mdr2^−/−^ mice.

### BBR modifies the bile acid metabolism in Mdr2^−/−^ mice

**Fig. 6:**
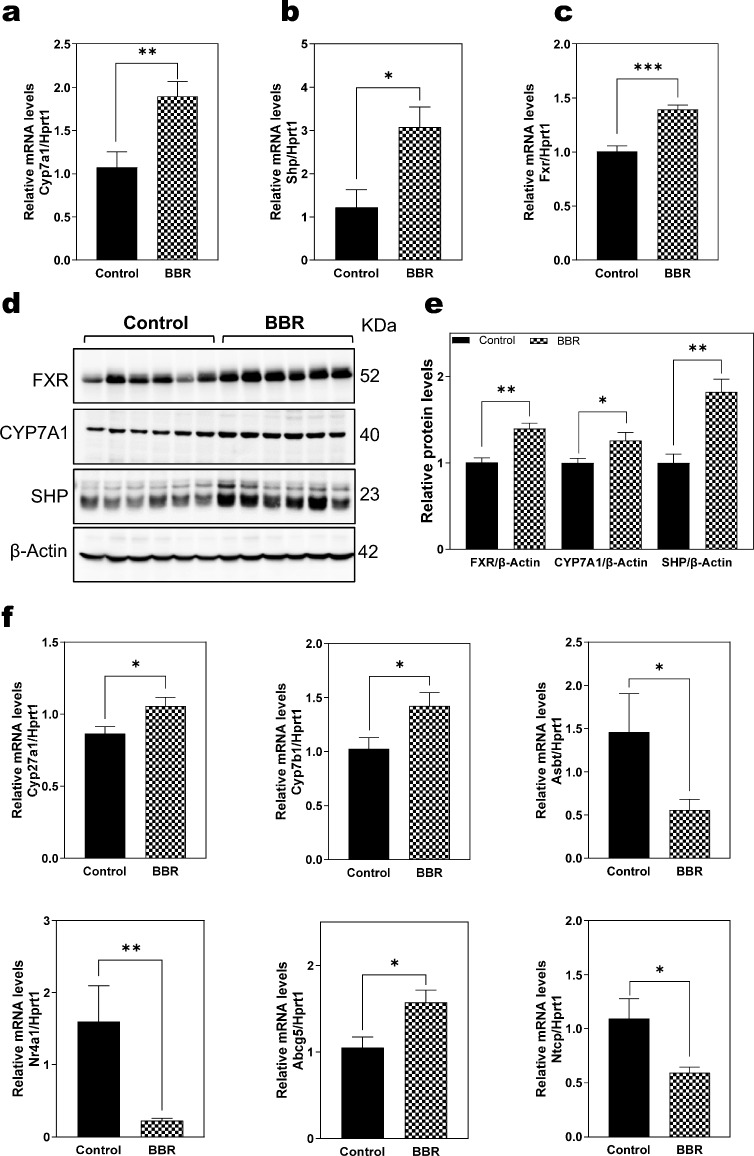
Effect of BBR on bile acid metabolism in Mdr2^−/−^ mice. **a–c, f** Relative mRNA levels of key genes involved in bile acid metabolism in the liver, including Cyp7a1, Shp (Nr0b2), Fxr(Nr1h4), Cyp27a1, Cyp7b1, Asbt, Nr4a1, Abcg5, and Ntcp. The mRNA levels were determined by qRT–PCR and normalized with HPRT1 as an internal control. **d** Representative immunoblot images of FXR, SHP, CYP7A1 and β-Actin are shown. **e** The relative protein levels of FXR, SHP, and CYP7A1 were calculated using β-actin as a loading control. Data are expressed as the mean ± SEM. Statistical significance relative to Control: *p < 0.05, **p < 0.01, ***p < 0.001 (n = 9–12)

Bile acid (BA) synthesis and transport are crucial for regulating the amount of BA in circulation [6]. As shown in Additional file 1: Fig. S11, the heatmap from RNA-seq analysis showed the key gene in primary bile acid biosynthesis, Cyp7a1 (Cholesterol 7 alpha-hydroxylase), the rate-limiting enzyme in classical pathways of BA synthesis, was significantly increased by BBR treatment in Mdr2^−/−^ mice. BBR also modulated the expression of genes involved in hepatic transporters and nuclear receptors, such as Abcg5, Asbt (Slc10a2), Shp, farnesoid X receptor α (Fxrα), etc. To further confirm the results of RNA-seq data, we measured the expression of the major genes using real-time RT-PCR. As shown in Fig. 6a–c, BBR significantly increased the mRNA expression level of Cyp7a1, Shp, and Fxrα. Western-blot analysis further showed that the protein expression levels of CYP7A1, SHP, and FXRα were significantly increased in Mdr2^−/−^ mice treated with BBR (Fig. 6d–e). As shown in Fig. 6f, the mRNA expression level of Cyp27a1, Cyp7b1, and Abcg5 were also increased in Mdr2^−/−^ mice by BBR, while the expression of Asbt and Nr4a1 were decreased by BBR. Although RNA-seq data did not identify significant changes in Ntcp (Slc10a1), the real-time PCR analysis showed that BBR was able to reduce the expression of Ntcp.

### BBR restores the BA homeostasis in Mdr2^−/−^ mice

**Fig. 7:**
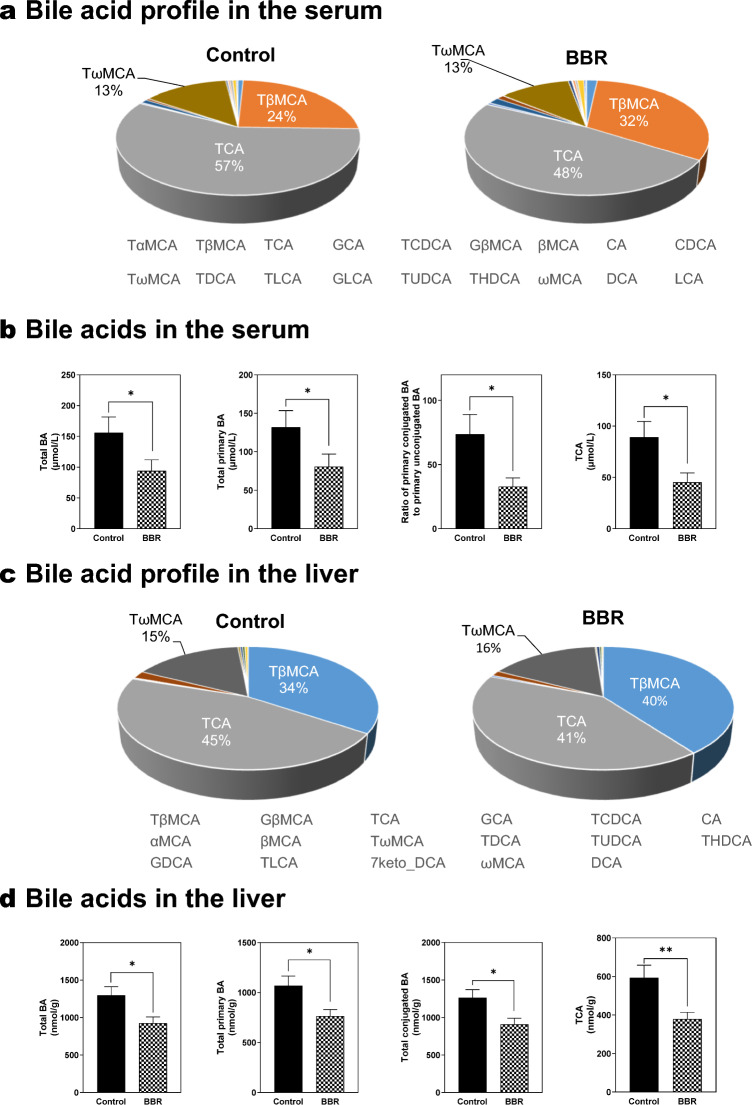
Impact of BBR on bile acid homeostasis in Mdr2^−/−^ mice. Serum and liver tissues were processed for bile acids (BA) analysis using liquid chromatography-tandem mass spectrometry (LC–MS/MS). **a** BA composition profile in the serum, expressed as a percentage of total BA. **b** Total BA, total primary BA, the ratio of primary conjugated BA to primary unconjugated BA, and TCA in the serum. **c** BA composition profile in the liver, expressed as a percentage of total BA. **d** Total BA, total primary BA, total conjugated BA, and TCA in the liver. Data are expressed as the mean ± SEM. Statistical significance relative to control: *p < 0.05, **p < 0.01 (n = 9–12)

Previous studies have reported that interruption of the enterohepatic circulation of BA protects against BA-mediated cholestatic liver and bile duct injury [26, 27]. Our recent study indicated that BBR restored the BA homeostasis in MASH mice [16]. To examine the effect of BBR on BA profile in Mdr2^−/−^ mice, we measured more than 30 different BAs in the serum, liver, intestine, and feces using LC–MS/MS. In the serum, as shown in Fig. 7a, BBR modulated the BAs composition by slightly reducing the percentage of taurocholic acid (TCA) in total BAs (from 57 to 48%) and increasing the percentage of tauro-β-muricholic acid (TβMCA) (from 24 to 32%). Among the individual BAs, the levels of TCA and tauro-ω-muricholic acid (TωMCA) were significantly decreased by BBR (Additional file 2: Table S2, Fig. 7b). The results further showed that BBR was able to decrease the total BAs, total primary BAs, the ratios of primary conjugated BAs to primary unconjugated BAs, etc. (Additional file 2: Table S3). In the liver, BBR slightly modulated the BAs composition by regulating the composition of TCA and TβMCA (Fig. 7c). As shown in Additional file 2: Table S4, Fig. 7d, the levels of TCA were significantly reduced by BBR. In addition, BBR was able to decrease the total BAs, total primary BAs, total conjugated BAs, etc. (Additional file 2: Table S5, Fig. 7d). In the intestine, as shown in Additional file 1: Fig. S12a, BBR modified the BA composition by slightly reducing the percentage of TCA in total BAs (from 37 to 30%). The total BAs, total primary BAs, total conjugated BAs, and TCA were also reduced by BBR (Additional file 1: Tables S6, S7 and Fig. S12b). In the feces, as shown in Additional file 1: Fig. S12c, the BA composition remained unchanged after BBR treatment. However, BBR was able to increase the total BAs, total secondary BAs, TCA, etc. (Additional file 2: Tables S8, S9 and Additional file 1: Fig. S11d). Interestingly, lithocholic acid (LCA), which has high cytotoxicity [28], was significantly increased in fecal samples of BBR. However, LCA is hydrophobic and mostly remains in the fecal pellet.

### BBR enhances intestinal barrier function and reduces bacterial translocation in Mdr2^−/−^ mice

**Fig. 8:**
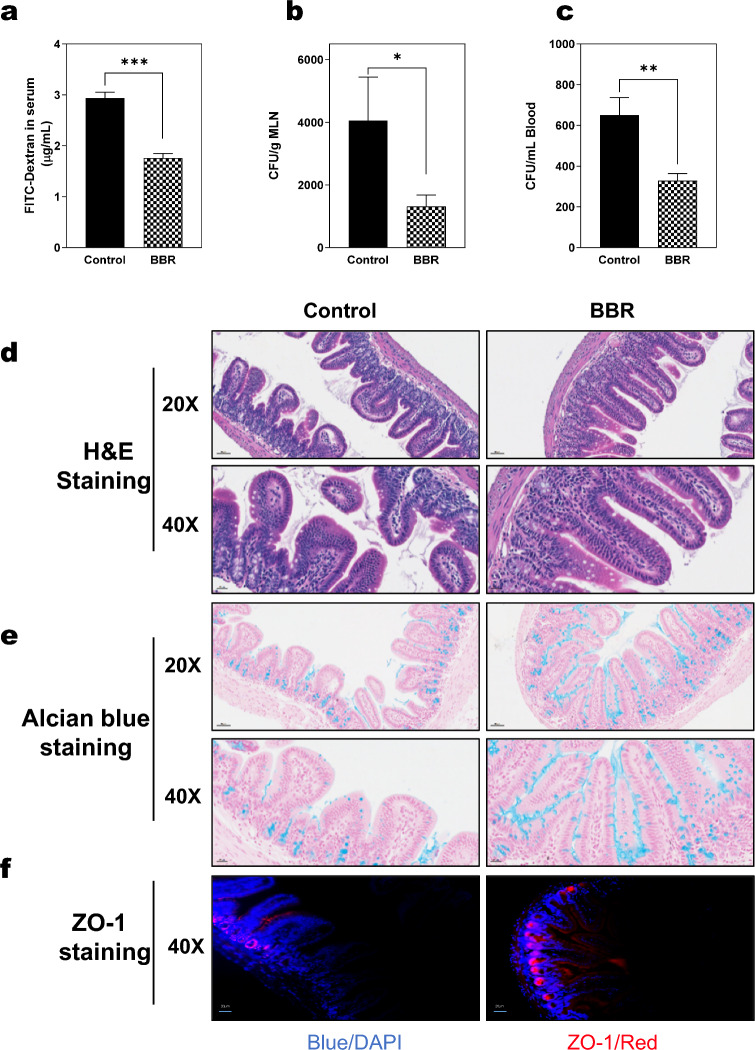
Impact of BBR on intestinal barrier function and bacterial translocation in Mdr2^−/−^ mice. **a** Serum levels of FITC-Dextran levels, a marker of intestinal permeability. **b-c** Colony-forming units (CFUs) of bacteria isolated from MLNs (mesenteric lymph node) and blood, respectively. **d** Representative images of H&E staining of small intestine sections (scale bar, 50 µm for 20 × and 20 µm for 40 × magnification). **e** Representative images of Alcian blue staining of small intestine sections (scale bar, 50 µm for 20 × and 20 µm for 40 × magnification).** f** Representative images of ZO-1 immunofluorescence (IF) staining of small intestine sections (scale bar, 20 µm for 40 × magnification). Data are expressed as the mean ± SEM. Statistical significance relative to Control: *p < 0.05, **p < 0.01, ***p < 0.001 (n = 9–12)

Compromised intestinal barrier integrity leads to bacterial migration to the liver, other organs and the bloodstream, causing systemic inflammation [7]. As shown in Fig. 8A, BBR treatment significantly reduced gut permeability in Mdr2^−/−^ mice, indicated by lower serum levels of fluorescein isothiocyanate (FITC)-Dextran. Additionally, bacteria presence in the mesenteric lymph nodes (MLNs) and blood was investigated. Notably, BBR treatment markedly decreased bacterial translocation to the MLNs (Fig. 8b). This reduction was consistent with fewer bacteria observed in the blood of BBR-treated Mdr2^−/−^ mice compared to untreated Mdr2^−/−^ mice (Fig. 8c). H&E staining revealed that Mdr2^−/−^ mice treated with BBR exhibited less lymphatic vessel dilation and inflammatory cell infiltration in their small intestines compared to untreated Mdr2^−/−^ mice (Fig. 8d). Additionally, BBR treatment was associated with increased mucus layer thickness, as evidenced by enhanced mucin-2 expression identified through Alcian blue staining (Fig. 8e). Immunofluorescence staining of the tight junction protein ZO-1 showed that BBR restored the integrity of tight junctions in the small intestines of these mice (Fig. 8f). Gene expression analysis further confirmed BBR's anti-inflammatory effects, with reduced mRNA levels of several inflammation-related genes (Mcp-1, Cd11b, Il-1β, Vcam-1, Il-1α, Cxcl1) in the small intestine of BBR-treated mice (Additional file 1: Fig. S13a). Notably, the expression levels of Asbt, Chop, and the LncRNA H19 were also decreased in these mice following BBR treatment (Additional file 1: Fig. S13b).

### BBR accumulates in the intestinal tract and modifies gut microbiome in Mdr2^−/−^ mice

In light of BBR's beneficial effects on the liver and intestine in Mdr2^−/−^ mice, we investigated its tissue distribution across various organs. After administering a single 50 mg/kg dose of BBR to these mice, we collected blood and major tissues at different time points for analysis (Additional file 2: Table S10). As shown in Additional file 1: Fig. S14a, the concentration of BBR in the serum was decreased gradually over time in Mdr2^−/−^ mice. Notably, BBR concentration was ~ 100-fold higher in the colon, intestine, and stomach, compared in the liver, kidney, heart, lung, brain, and spleen (Additional file 1: Fig. S14b). This suggests that BBR mitigates cholestatic liver injury by modulating the gut-liver axis, an important pathway influenced by the gut microbiome, which is crucial for BA deconjugation and excretion. Considering the role of the gut microbiome in PSC [1, 2, 6], we performed 16S rRNA gene sequencing. The analysis revealed that while Alpha diversity (gut microbiota structure within a community) showed no significant changes with BBR treatment (Additional file 1: Fig. S15a), Beta diversity (the comparison of gut microbiota structure between communities) displayed significant changes, though without a dose-dependent effect (Additional file 1: Fig. S15b and Table S11). The microbial composition in BBR-treated Mdr2^−/−^ mice showed a slight increase in *Bacteroidetes* (69% vs. 65% in the control group) and a decrease in *Firmicutes* (30% vs. 34% in the control group), indicating that BBR could modify the microbial structure (Additional file 1: Fig. S16).

## Discussion

Cholestatic liver diseases, marked by reduced bile flow, manifest through inflammation, ductular proliferation, and fibrosis. Patients with PSC and concurrent inflammatory bowel diseases (IBDs) face a significantly higher risk of developing cholangiocarcinoma and colorectal cancer [1, 4]. Currently, there is no effective medication for improving transplant-free survival in these cases. Liver transplantation is the only definitive treatment for PSC, though it carries a high risk of disease recurrence [2, 29, 30]. Recent studies suggest that targeting pathways, such as BA synthesis and transport, hepatic inflammation, mitochondrial respiration, oxidative stress, intestinal inflammation, and gut microbiota, could provide new therapeutic strategies for cholestatic liver diseases [26, 27, 31–35].

BBR has long been used in Asia as an anti-bacterial medicine. Clinical and preclinical studies highlight its potential in treating metabolic diseases by modulating various molecular targets, including transcription factors, cell survival/proliferative proteins, enzymes, metastatic/invasion molecules, growth factors, platelet activation, inflammatory cytokines, apoptotic proteins, protein kinases, receptors, and the others [14, 36]. There are a considerable number of studies demonstrating that BBR has preventive or therapeutic effects on various liver diseases, such as hepatitis, MAFLD, and liver fibrosis [11, 37–42]. However, its impact on cholestatic liver injury remains unexplored. To elucidate the therapeutic effect and potential mechanisms of BBR on cholestatic liver injury, we conducted a series of analyses, including histological imaging, biochemical analysis, molecular biology, and RNA-seq transcriptome analysis. Using bioinformatic tools, we identified differentially expressed genes regulated by BBR followed by GO, KEGG pathway, and functional category analysis. The findings of this study strongly suggest that BBR is potentially effective in treating cholestatic liver injury. The major mechanisms underlying BBR's beneficial effects include reducing bile duct injury and hepatic fibrosis, alleviating hepatic inflammation and ER stress, restoring BA homeostasis, and improving intestinal barrier function as well as modulating gut microbiome.

PSC is an inflammatory liver disease often associated with severe cholestatic liver injury [43]. BBR is known for its potent anti-inflammatory activities in liver disease [16, 17]. Key pathways, such as NF-κB signaling pathway, MAPK pathways, and oxidative phosphorylation, are involved in inflammation-driven cholestatic liver injury [8, 23, 44, 45]. Our RNA-seq gene analysis and pathway profiling showed that BBR significantly reduced inflammation in Mdr2^−/−^ mice. This reduction is achieved through BBR's ability to inhibit inflammatory macrophage infiltration in the liver, which is evident from the decreased expression of various chemokines, cytokines, and cell surface adhesion molecules (Fig. 4, Additional file 1: Fig. S6). Additionally, BBR modulates NF-κB signaling, the MAPK signaling pathway, and oxidative phosphorylation (Additional file 1: Fig. S7–S9).

CCL2/MCP-1 chemokines, produced by fibroblasts, activated cholangiocytes, resident macrophages, and endothelial cells, play a crucial role in the inflammatory response. A recent study suggests that targeting the CCR2/CCL2 axis can limit monocyte recruitment and reduce fibrosis and cholestasis, offering a potential treatment approach for PSC [46]. In line with these findings, our study demonstrates that BBR suppresses the hepatic expression of CCR2 and CCL2. Furthermore, the ER stress response, a key factor in inflammation and metabolic disorders, is significantly modulated by BBR [24, 47, 48]. Disruptions in ER homeostasis activate the UPR, leading to inflammation and cell injury. The IRE1, protein kinase RNA-like ER kinase (PERK), and ATF6 pathways are the three major branches of the UPR [24]. BBR has been previously shown to inhibit HIV protease inhibitor-induced ER stress in macrophages and inhibit free fatty acid and LPS-induced inflammation via modulating the PERK-ATF4-CHOP signaling pathway in macrophages and hepatocytes [17, 18]. Consistently, our current study found that BBR significantly reduced ER stress in Mdr2^−/−^ mice, particularly inhibiting the PERK-ATF4-CHOP pathway (Fig. 5 and Additional file 1: Fig. S10).

BA homeostasis is crucial in managing cholestatic liver diseases [3, 49]. BAs are synthesized in hepatocytes and immediately secreted into bile through the bile duct. The majority of BAs are reabsorbed in the terminal ileum and transported back to the liver through portal vein. The enterohepatic circulation of BA is an important physiological process, making the synthesis and transport of BAs vital targets for cholestatic liver injury [50, 51]. Our studies show that BBR can restore BA homeostasis by modulating key enzymes, nuclear receptors, and hepatic transporters involved in BA synthesis and transport (Fig. 6 and Additional file 1: Fig. S11). Elevated serum BA levels, particularly total, primary, conjugated BAs, including TCA, were common in PSC patients [52–54]. The current study showed that BBR treatment in Mdr2^−/−^ mice significantly reduced these BA levels in serum, liver, and small intestine while increasing fecal BA output without causing diarrhea (Fig. 7 and Additional file 1: Fig. S12). This suggests BBR's role as a differential BA transport inhibitor, indicated by reduced Ntcp and Asbt expression (Fig. 6). Furthermore, BBR has been shown to influence key regulators of BA homeostasis significantly. In our study, BBR increased the expression of Fxrα, a crucial regulator in BA homeostasis, and increased the expression of Shp, which represses Cyp7a1 by inhibiting LRH-1 activity [55, 56]. However, the RNA-seq data showed BBR had no significant effects on LRH-1, but upregulated both Cyp7a1 and Cyp27a1 levels in the liver (Fig. 6). These results suggest the potential compensatory mechanisms to counteract the inhibition of BA up taking in Mdr2^−/−^ mice with BBR treatment. Although FXR agonists and ASBT inhibitors have been tested in clinical trials for various liver diseases, the potential to treat PSC remains uncertain. Our previous studies reported that increased primary conjugated BA is responsible for cholestatic liver injury and liver fibrosis via activating sphingosine-1 phosphate receptor 2 (S1PR2), which can upregulate lncRNA H19 in Mdr2^−/−^ mice [57–60]. Our recent study showed that BBR reduced the expression of H19 in a MASH mouse model [16]. Consistently, in this study, our results showed that H19 was inhibited by BBR treatment in Mdr2^−/−^ mice.

Recent clinical studies have established a link between disrupted intestinal barrier function, bacterial translocation, and the progression of cholestatic liver diseases, such as PSC and PBS [61]. Specifically, in Mdr2^−/−^ mice, impairment in intestinal barrier function has been observed, including diminished tight junction protein expression, reduced mucus layers, increased permeability, and enhanced bacterial translocation [9]. Our previous study has reported that ER stress-induced activation of CHOP leads to disruption of intestinal barrier function, bacterial translocation, activation of inflammation, and eventually results in fibrosis in the liver [7]. In line with these findings, our current study demonstrates that BBR effectively decreased CHOP expression in both the liver and intestine (Fig. 5d and Additional file 1: Fig. S13b), suggesting its potential to mitigate these pathological processes. Moreover, recent studies reported that H19 exacerbates intestinal barrier dysfunction by inhibiting autophagy and impairing goblet and Paneth cell functions [62, 63]. Consistent with this, our results show that BBR significantly inhibits H19, correlating with restored epithelial barrier function as evidenced by increased expression of mucin-2 and ZO-1 (Figs. 8e-f & Additional file 1: Fig. S13b).

A previous study using hamsters found that orally administered BBR predominantly accumulates in the gut rather than in circulation, significantly affecting both gut and circulatory metabolites despite low serum levels [64]. Our study aligns with these findings, showing that BBR concentrations are highest in the stomach, intestine, and colon and relatively lower in the liver, kidney, heart, lung, brain, and spleen. This suggests that BBR mitigates cholestatic liver injury by modulating the gut-liver axis (Additional file 1: Fig. S14). It is well established that the gut microbiota regulates BA composition and levels, particularly in PSC. BBR has been reported to have antidiabetic effects by modulating the gut microbiome [10, 15]. Our current study further indicates that BBR alters the gut microbiota composition in Mdr2^−/−^ mice. Specifically, BBR increased the relative abundance of *Bacteroidetes* and decreased that of *Firmicutes* (Additional file 1: Fig. S15), which is significant as bacteria in *Firmicutes* are known for high bile salt hydrolase (BSH) activity, promoting BA deconjugation and fecal excretion [65, 66]. This alteration in microbiota composition aligns with the interplay between intestinal microbiota and BAs, where each influence the other.

## Conclusion

**Fig. 9:**
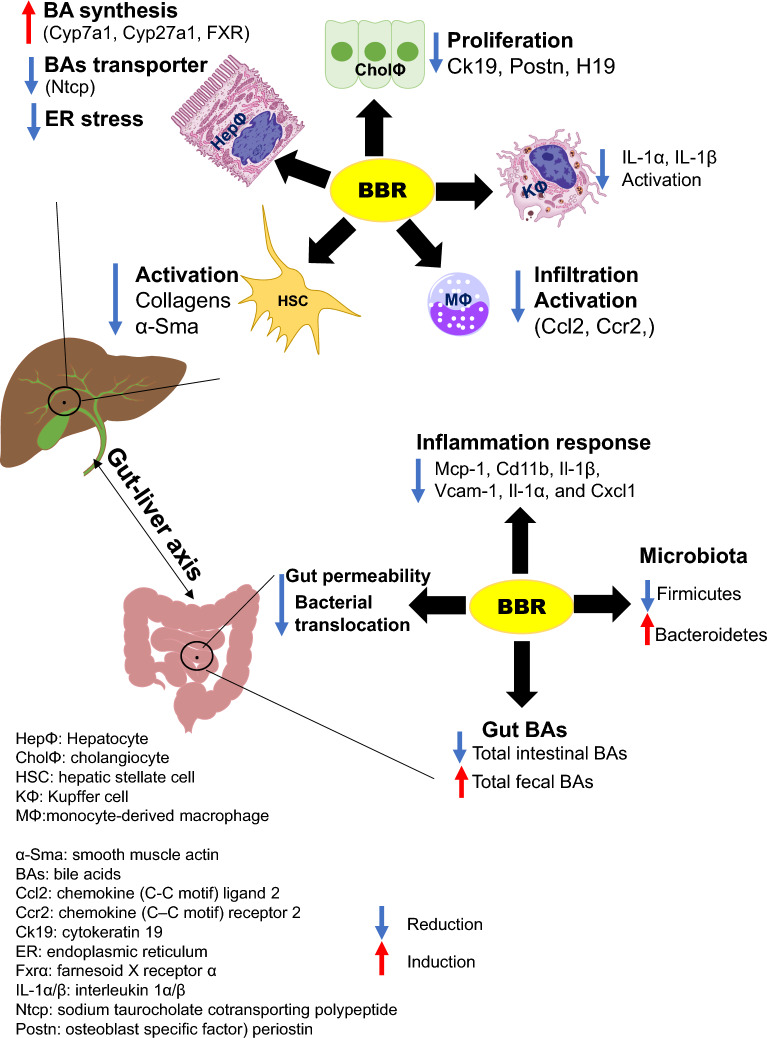
Schematic Representation of BBR’s Potential Mechanisms in Alleviating Cholestatic Liver Injury. This diagram illustrates the proposed molecular and cellular mechanisms through which BBR mitigates cholestatic liver injury in a mouse model of sclerosing cholangitis. It visually summarizes the pathways and interactions influenced by BBR treatment, highlighting its multifaceted role in addressing liver disease pathology

In summary, our study sheds light on the potential mechanisms by which BBR attenuates cholestatic liver injury in a PSC mouse model. As illustrated in Fig. 9, BBR can directly or indirectly target various liver cells, including hepatocytes, macrophages, stellate cells, and cholangiocytes, modulating multiple pathways related to bile duct injury, fibrosis, inflammation, ER stress, and BA metabolism and transport in the gut-liver-axis. Furthermore, BBR enhances intestinal barrier function and reduces bacterial translocation, while also restoring BA homeostasis and gut microbiota. These findings suggest that BBR has potential as a pharmacological treatment for cholestatic liver injury such as PSC.

## Materials and methods

### Reagents

Berberine chloride hydrate (BBR) was purchased from Sigma (St. Louis, MO, USA, Cat #14050). Common laboratory chemicals were purchased from Sigma Aldrich (St. Louis, MO, USA). All antibodies used in this study are listed in Additional file 2: Table S1.

### Animal experiments

FVB Mdr2^−/−^ mice (100 days old, both sexes, n = 9–12) were originally obtained from Dr. Gianfranco Alpini (Texas A&M HSC College of Medicine). Mdr2^−/−^ mouse (C57/BL6 background) is a kind gift from Dr. Daniel Goldenberg at the Department of Pathology, Hadassah-Hebrew University Medical Center, Jerusalem, Israel. Mice were randomly divided into the vehicle control group and BBR group. Mice were treated with BBR (50 mg/kg) or vehicle (0.5% carboxyl methyl cellulose sodium solution) by intragastric administration once daily for 8 weeks. All mice were housed in a 12 h light/12 h dark cycle with a controlled room temperature between 21 and 23 °C and free access to water. All the experimental procedures were performed according to protocols approved by the Richmond VA Medical Center and Virginia Commonwealth University Institutional Animal Care and Use Committee. All animal experiments were performed in accordance with institutional guidelines for ethical animal studies. At the end of the experiment, mice were weighed and anesthetized by exposure to inhaled isoflurane. The blood was collected by cardiac puncture. The serum was collected and stored at − 80 °C for later analysis. After euthanasia, the liver and small intestine were collected for histological analysis, RNA profiling, and Western blot analysis. Fecal samples were collected for 16S rRNA gene sequencing to measure the gut microbiome.

### RNA sequencing (RNAseq) and bioinformatic analysis

Total liver RNA was isolated using Chemagic Prepito®-D Nucleic Acid Extractor (PerkinElmer, Waltham, MA, USA) with a Prepito RNA kit (PerkinElmer, USA). The RNAseq with ribosomal RNA (rRNA) depletion was done by Genewiz Company using the Illumina Hiseq® X platform (Genewiz Co., South Plainfield, NJ, USA). Sequencing reads were trimmed and filtered using bbduk to remove adapters and low-quality reads. Reads from mouse samples were mapped to Ensembl GRCm38 transcripts annotation (release 82), using RSEM. Gene expression data normalization and differential expression analysis were performed using the R package edgeR. Significantly up- or downregulated genes were determined as fold change ≥ 2 and *p*-value < 0.05. Hierarchical clustering was performed to show distinguishable mRNA expression profiles among the samples (Heatmap was plotted by http://www.bioinformatics.com.cn, an online platform for data analysis and visualization). The volcano graph and heatmaps were created to visualize significantly dysregulated mRNAs using GraphPad Prism (version 8; GraphPad Software Inc., San Diego, CA, USA). Gene Ontology (GO) analysis was used to investigate three functionality domains: biological process (BP), cellular component (CC), and molecular function (MF) using DAVID (Database for Annotation, Visualization, and Integrated Discovery) v6.8 (https://david.ncifcrf.gov/). Pathway analysis was performed to functionally analyze and map genes to Kyoto Encyclopedia of Genes and Genomes (KEGG) pathways (https://pathview.uncc.edu/).

### Serum biochemical analysis and hepatic hydroxyproline content measurement

The serum levels of ALP, AST and ALT, total triglyceride (TG), total cholesterol (TC), very-low-density lipoprotein (VLDL), and ALB were determined using the Alfa Wassermann Vet ACE Axcel® System with commercially available assay kits (Alfa Wassermann diagnostic technologies, NJ, USA). To quantify liver fibrosis, hepatic hydroxyproline was measured using the Hydroxyproline Assay kit (Sigma Aldrich, MO, USA) according to the manufacturer's instructions.

### Histological and immunohistochemical staining

Liver tissues were processed for hematoxylin and eosin (H&E) staining and immunohistochemistry (IHC) staining for CK-19 and Ki67 at the Mouse Model Core at the VCU Massey Cancer Center (Richmond, VA, USA). Picro Sirius Red Staining was performed using the commercial Kit (Abcam, USA) with the paraffin-embedded tissue sections according to the manufacturer's instructions. Small intestine tissues were processed for H&E staining. Alcian blue staining was performed using the Alcian blue Stain Kit (Abcam, USA). Immunofluorescence staining of ZO-1 was performed with the paraffin-embedded tissue sections according to the manufacturer's instructions. All the stained slides were scanned using a Vectra Polaris Automated Quantitative Pathology Imaging System (Akoya Biosciences, MA, USA), and the images were captured using Phenochart software (Akoya Biosciences, MA, USA).

### Bile acid (BA) analysis

The serum, liver tissues, intestine contents, and colon feces were processed for BA analysis, as described previously [16]. The composition and levels of BAs in serum, liver, intestine and fecal samples were measured using a Shimadzu liquid chromatography/tandem mass spectrometric (LC–MS/MS) 8600 system as described previously [16]. Data were collected and processed using Lab Solutions software.

### Tissue distribution of BBR

Mdr2^−/−^ mice were treated with BBR (50 mg/kg) by intragastric administration after a 12-h fast. Blood, heart, lung, liver, kidney, brain, spleen, stomach, intestine, colon, and feces were collected after 3, 6, 9, and 12 h of BBR treatment, respectively. The contents of BBR in serum and tissues were analyzed using LC–MS/MS.

A reliable LC–MS/MS method was developed and validated to quantify BBR, using L-tetrahydropalmatine as the internal standard (IS). To quantify BBR in the serum, serum samples and IS were incubated with acetonitrile/methanol/water l (1/1, v/v) in a 1.5 mL vial. For quantification of BBR in the spleen, lung, kidney, heart, stomach contents, intestine contents, and feces, tissue samples were incubated with acetonitrile/methanol (1/1, v/v) in a 2 mL vial with beads. The homogenized samples and IS were mixed with acetonitrile/methanol/water (1/1, v/v) in a 1.5 mL vial. After centrifugation at 12,000 × g for 2 min at room temperature, the supernatant was filtered through 0.2 µm PTFE membrane, and 2 µL aliquots were injected into the LC–MS/MS system. The analyte was separated on a C18 reverse phase column and analyzed in the multiple reaction monitoring (MRM) mode using ESI with positive ionization, m/z 335.9 → 320.1 for BBR and m/z 355.9 → 192.2 for IS. Mobile phase A was 0.05% acetic acid in water, while mobile phase B was acetonitrile. The gradient was optimized at 30% to 75% B in 2 min and then maintained 75% B for 0.5 min. The column was equilibrated with 30% B for 1.5 min. Data were collected and processed using Lab Solutions software.

### Quantitative RT-PCR

Total liver RNA was isolated using Chemagic Prepito®-D Nucleic Acid Extractor (PerkinElmer, USA) with Prepito RNA kit (PerkinElmer, USA). cDNA synthesis and Quantitative RT-PCR analysis of relative mRNA expression levels of target genes were previously described [16]. Primer sequences will be provided upon request.

### Immunoblotting analysis

Total proteins were prepared using cold RIPA buffer. Nuclear proteins were isolated, as previously described. Protein concentration was measured using the Bio-Rad Protein Assay reagent. Proteins were resolved on 10% SDS-PAGE and transferred to nitrocellulose membranes (Thermo, Waltham, MA, USA). 5% milk was used to block the background. The target proteins were probed with the specific primary antibodies and detected using HRP-conjugated secondary antibodies and ECL reagents (Thermo, USA). Images were captured using the Bio-Rad Gel Doc XR + Imaging System (Hercules, CA, USA). The density of immunoblotted bands was analyzed using BioRad Image Lab computer software and normalized with histone 3 or β-Actin.

### FITC-DEXTRAN permeability and bacterial translocation assay

FITC-Dextran solution (100 mg/mL) was prepared in PBS. FITC-Dextran was administered to mice by oral gavage (600 mg/kg) and blood samples were taken after 4 h. The serum concentration of FITC-dextran was measured using Victor Multilabel Plate Counter (PerkinElmer, Waltham, MA) with an excitation wavelength of 490 nm and an emission wavelength of 530 nm. Blood and mesenteric lymph nodes (MLNs) were harvested in sterile conditions. Blood and homogenized MLNs were diluted in series and plated on Blood Agar Plates. After 72 h incubation at 37 °C in aerobic conditions, colony-forming units (CFUs) were counted and calculated.

### Microbiota analysis

Fecal samples of Mdr2^−/−^ mice treated with BBR 50 mg/kg or 100 mg/kg for 8 weeks were collected for 16S rRNA gene sequencing. Extraction, library preparation, sequencing, and analysis were performed at Rutgers Center for Microbiome Analysis Core, New Jersey Institute for Food, Nutrition and Health. All DNA samples were quantified using the Qubit 1 × dsDNA HS assay kit (Thermo Fisher Scientific), which measured DNA concentration based on the fluorescence intensity of a fluorescent dye binding to double-stranded DNA. DNA integrity was assessed using agarose gel electrophoresis.

### Statistical analysis

Data are expressed as the mean ± SEM from at least three independent experiments. The student's t-test was used to analyze the difference between the two groups by GraphPad Prism (version 8; GraphPad Software Inc., San Diego, CA). A *p*-value < 0.05 was considered statistically significant.

### Supplementary Information

**Additional file 1: Fig. S1.** Impact of BBR on body weight and serum albumin levels in FVB Mdr2^-/-^ mice and cholestatic liver injury in C57/BL6 Mdr2^-/-^ mice. Mdr2^-/-^ mice with FVB background (Control) and Mdr2^-/-^ mice with C57BL/6 background (Control BL) were treated with vehicle or BBR (50 mg/kg) via oral gavage once daily for 8 weeks, respectively. a Body weight change during the BBR treatment period of 8 weeks in FVB Mdr2^-/-^ mice. b Serum albumin levels in FVB Mdr2^-/-^ mice. c Liver functional enzyme levels in C57/BL6 Mdr2^-/-^ mice. d Representative images of hematoxylin and eosin (H&E) staining of the liver slides (scale bar, 50 µm for 20x, 20 µm for 40× magnification) in Mdr2^-/-^ BL mice. Data are expressed as the mean ± standard error of the mean (SEM). Statistical significance relative to Control BL: *p < 0.05 (n=9-12). **Fig. S2.** Comparative analysis of differentially expressed genes (DEGs) in experimental groups. a Hierarchical clustering heatmaps for DEGs in FVBWT, Mdr2^-/-^ and Mdr2^-/-^ mice treated with BBR. RNA-seq data were normalized using a Z-score for tag counts, with red and blue colors representing high and low gene expression, respectively. b Volcano plots for the Mdr2^-/-^ vs. WT group comparison. Red dots represent upregulated genes, green dots represent downregulated genes, and black dots represent genes not differentially expressed. c Venn diagram illustrating the overlap of DEGs between the two comparisons: Mdr2^-/-^ vs. WT and BBR-treated Mdr2^-/-^ vs. Mdr2^-/-^ Control. In Mdr2^-/-^ vs. WT, there were a total of 1937 DEGs, including 1260 upregulated and 677 down-regulated genes. In BBR-treated Mdr2^-/-^ vs. Mdr2^-/-^ Control, there were a total of 587 DEGs, comprising 300 upregulated and 287 down-regulated genes. A total of 373 DEGs were common between the two comparisons. **Fig. S3.** Ingenuity pathway analysis (IPA) in experimental groups. The DEG data set with FC ≥2 and p-value <0.05 was used for IPA analysis. The top 10 activated pathways in Mdr2^-/-^ control mice compared to WT mice and the top 10 inhibited pathways in BBR-treated Mdr2^-/-^ mice compared to Mdr2^-/-^ control mice are shown. **Fig. S4.** Impact of BBR on hepatic fibrosis. a Representative images of liver sections stained with Picro-Sirius Red and CK19 IHC (scale bar, 100 µm for 10× magnification) and processed images for quantification. b Hepatic hydroxyproline levels. Data are expressed as the mean ± SEM. Statistical significance relative to control: *p < 0.05 (n=9-12). **Fig. S5.** Impact of BBR on genes associated with hepatic fibrosis in Mdr2^-/-^ mice. a Representative heatmap of key genes involved in hepatic fibrosis in the liver, comparing the BBR-treated group with the control group. The RNA-seq data were normalized using a Z-score for tag counts, with red and blue colors denoting up- and down-regulated gene expression, respectively. b Relative mRNA expression levels of fibrosis-related genes (Pai1,Col12a1, Sox9, Egr1, Egr2, Egr3, Hbegf, Cyr61, and P4ha1), normalized against HPRT1 as an internal control. Data are expressed as the mean ± SEM. Statistical significance relative to control: *p < 0.05, **p < 0.01, ***p < 0.001(n=9-12). **Fig. S6.** Impact of BBR on genes associated with inflammation in Mdr2^-/-^ mice. Representative heatmap depicting the expression of key genes involved in hepatic inflammation, comparing the liver tissues of Mdr2^-/-^ mice treated with BBR to the control group. The RNA-seq data were normalized using a Z-score, with red indicating upregulated gene expression and blue indicating downregulated gene expression. **Fig. S7.** Impact of BBR on NF-kB signaling pathway. KEGG pathway analysis was performed on RNA-seq data to analyze functionally and map genes involved in the NF-kB signaling pathway. a NF-kB signaling pathway in Mdr2^-/-^ vs. WT. b NF-kB signaling pathway in Mdr2^-/-^ treated with BBR vs. Mdr2^-/-^ Control. Red and green colors indicate upregulated and downregulated gene expression, respectively. **Fig. S8.** Impact of BBR on MAPK signaling pathway. KEGG pathway analysis was performed on RNA-seq data to analyze functionally and map genes involved in the MAPK signaling pathway. a MAPK signaling pathway in Mdr2^-/-^ vs. WT. b MAPK signaling pathway in Mdr2^-/-^ treated with BBR vs. Mdr2^-/-^ Control. Red and green colors indicate upregulated and downregulated gene expression, respectively. **Fig. S9.** Impact of BBR on Oxidative phosphorylation pathway. KEGG pathway analysis was performed on RNA-seq data to analyze functionally and map genes involved in the Oxidative phosphorylation pathway. a Oxidative phosphorylation pathway in Mdr2^-/-^ vs. WT. b Oxidative phosphorylation pathway in Mdr2^-/-^ treated with BBR vs. Mdr2^-/-^ Control. Red and green colors indicate up- and down-regulated gene expression, respectively. **Fig. S10.** Impact of BBR on Protein processing in endoplasmic reticulum. RNA-seq data were performed to analyze functionally and map genes involved in the Protein processing in the endoplasmic reticulum pathway using KEGG. a Protein processing in endoplasmic reticulum pathway in Mdr2^-/-^ Control vs. WT. b Protein processing in endoplasmic reticulum pathway in Mdr2^-/-^ treated with BBR vs. Mdr2^-/-^ Control. Red and green colors indicate up- and down-regulated gene expression, respectively. **Fig. S11.** Impact of BBR on BA Metabolism. Representative heatmap of key genes involved in bile acid metabolism in the liver of BBR-treated vs. Control Mdr2^-/-^ mice. A Z-score is calculated for the RNA-seq data to normalize tag counts. Red and blue colors indicate up- and down-regulated gene expression, respectively. **Fig. S12.** Impact of BBR on bile acid homeostasis in Mdr2^-/-^ mice. The small intestine and feces were processed for BA analysis using LC-MS/MS. a BA composition profile in the small intestine is expressed as a percentage of total BA. b Total BA, total primary BA, total conjugated BA, and TCA in the small intestine. c BA composition profile in the feces is expressed as a percentage of total BA. d Total BA, total secondary BA, TCA, and LCA in the feces. Data are expressed as the mean ± SEM. Statistical significance relative to control: *p < 0.05 (n=9-12). **Fig. S13.** Effect of BBR on inflammation and ER stress in the intestine of Mdr2^-/-^ mice. Relative mRNA levels of key genes involved in inflammation and ER stress in the intestine were determined by real-time RT–PCR and normalized with HPRT1 as an internal control. a The relative mRNA levels of Mcp-1, Cd11b, Il-1β, Vcam-1, Il-1α, and Cxcl1. b Relative mRNA levels of Asbt, Chop and H19. Data are expressed as the mean ± SEM. Statistical significance relative to Control: *p < 0.05 (n=9-12). **Fig. S14.** Tissue distribution of BBR in Mdr2^-/-^ mice. Mdr2^-/-^ mice were treated with BBR (50 mg/kg, n = 3) by intragastric administration after a 12-h fast. Blood, spleen, brain, lung, heart, kidney, liver, stomach contents, intestine contents, and colon feces were collected at 3, 6, and 9 h post-treatment. The concentrations of BBR in the serum and various tissues were quantified using LC-MS/MS. a BBR concentration in the serum. b BBR concentration in the tissues. Data are expressed as the mean ± standard error of the mean (SEM). **Fig. S15.** Analysis of Fecal Microbiota Diversity in Mdr2^-/-^ Mice Treated with BBR. Fecal samples of FVB Mdr2^-/-^ mice treated with either 50 mg/kg or 100 mg/kg of BBR for 8 weeks were subjected to 16S rRNA gene sequencing to assess microbiota composition. a Alpha diversity of the fecal microbiota, presented through various metrics: Shannon Index (a), observed Amplicon Sequence Variants (ASVs) (b), Faith’s phylogenetic diversity (c), and evenness (d). b Beta diversity analysis using Principal Coordinates Analysis (PCoA) plots, which illustrate variations in microbial communities. These plots are based on different distance metrics: Bray-Curtis distance (a), Jaccard distance (b), Weighted UniFrac distance (c), and Unweighted UniFrac distance (d), with each plot depicting variations along two principal coordinates that account for most of the variation. **Fig. S16.** Influence of BBR on the Proportions of Firmicutes and Bacteroidetes in the Gut Microbiota of Mdr2^-/-^ mice. The pie chart shows the relative percentages of the Firmicutes and Bacteroidetes phyla in the gut microbiota of Mdr2^-/-^ mice. Comparative analysis is shown across three groups: control, BBR-treated at 50 mg/kg, and BBR-treated at 100 mg/kg. This visualization highlights the specific shifts in these major bacterial phyla due to BBR treatment.

**Additional file 2.** Supplement tables with captions.

## Data Availability

Detailed methods and datasets generated and/or analyzed during the current study are available in Additional file.

## Abbreviations

HSC: Hepatic stellate cells
lncRNA: Long non-coding RNA
PBC: Primary biliary cholangitis
PSC: Primary sclerosing cholangitis
S1PR2: Sphingosine-1 phosphate receptor 2
SHP: Short heterodimer partner
TCA: Taurocholate sodium
WT: Wild type
