# Supplementary material for: Therapeutic potential of berberine in attenuating cholestatic liver injury: insights from a PSC mouse model
Source: Cell Biosci. 2024 Jan 25;14:14. doi: 10.1186/s13578-024-01195-8 (PMC10809567; doi:10.1186/s13578-024-01195-8)
Supplement: Supplementary file 1 — Additional file 1: Fig. S1. Impact of BBR on body weight and serum albumin levels in FVB Mdr2-/- mice and cholestatic liver injury in C57/BL6 Mdr2-/- mice. Mdr2-/- mice with FVB background (Control) and Mdr2-/- mice with C57BL/6 background (Control BL) were treated with vehicle or BBR (50 mg/kg) via oral gavage once daily for 8 weeks, respectively. a Body weight change during the BBR treatment period of 8 weeks in FVB Mdr2-/- mice. b Serum albumin levels in FVB Mdr2-/- mice. c Liver functional enzyme levels in C57/BL6 Mdr2-/- mice. d Representative images of hematoxylin and eosin (H&E) staining of the liver slides (scale bar, 50 µm for 20x, 20 µm for 40× magnification) in Mdr2-/- BL mice. Data are expressed as the mean ± standard error of the mean (SEM). Statistical significance relative to Control BL: *p < 0.05 (n=9-12). Fig. S2. Comparative analysis of differentially expressed genes (DEGs) in experimental groups. a Hierarchical clustering heatmaps for DEGs in FVBWT, Mdr2-/- and Mdr2-/- mice treated with BBR. RNA-seq data were normalized using a Z-score for tag counts, with red and blue colors representing high and low gene expression, respectively. b Volcano plots for the Mdr2-/- vs. WT group comparison. Red dots represent upregulated genes, green dots represent downregulated genes, and black dots represent genes not differentially expressed. c Venn diagram illustrating the overlap of DEGs between the two comparisons: Mdr2-/- vs. WT and BBR-treated Mdr2-/- vs. Mdr2-/- Control. In Mdr2-/- vs. WT, there were a total of 1937 DEGs, including 1260 upregulated and 677 down-regulated genes. In BBR-treated Mdr2-/- vs. Mdr2-/- Control, there were a total of 587 DEGs, comprising 300 upregulated and 287 down-regulated genes. A total of 373 DEGs were common between the two comparisons. Fig. S3. Ingenuity pathway analysis (IPA) in experimental groups. The DEG data set with FC ≥2 and p-value <0.05 was used for IPA analysis. The top 10 activated pathways in Mdr2-/- control mice [file 13578_2024_1195_MOESM1_ESM.pptx]

## Slide 1
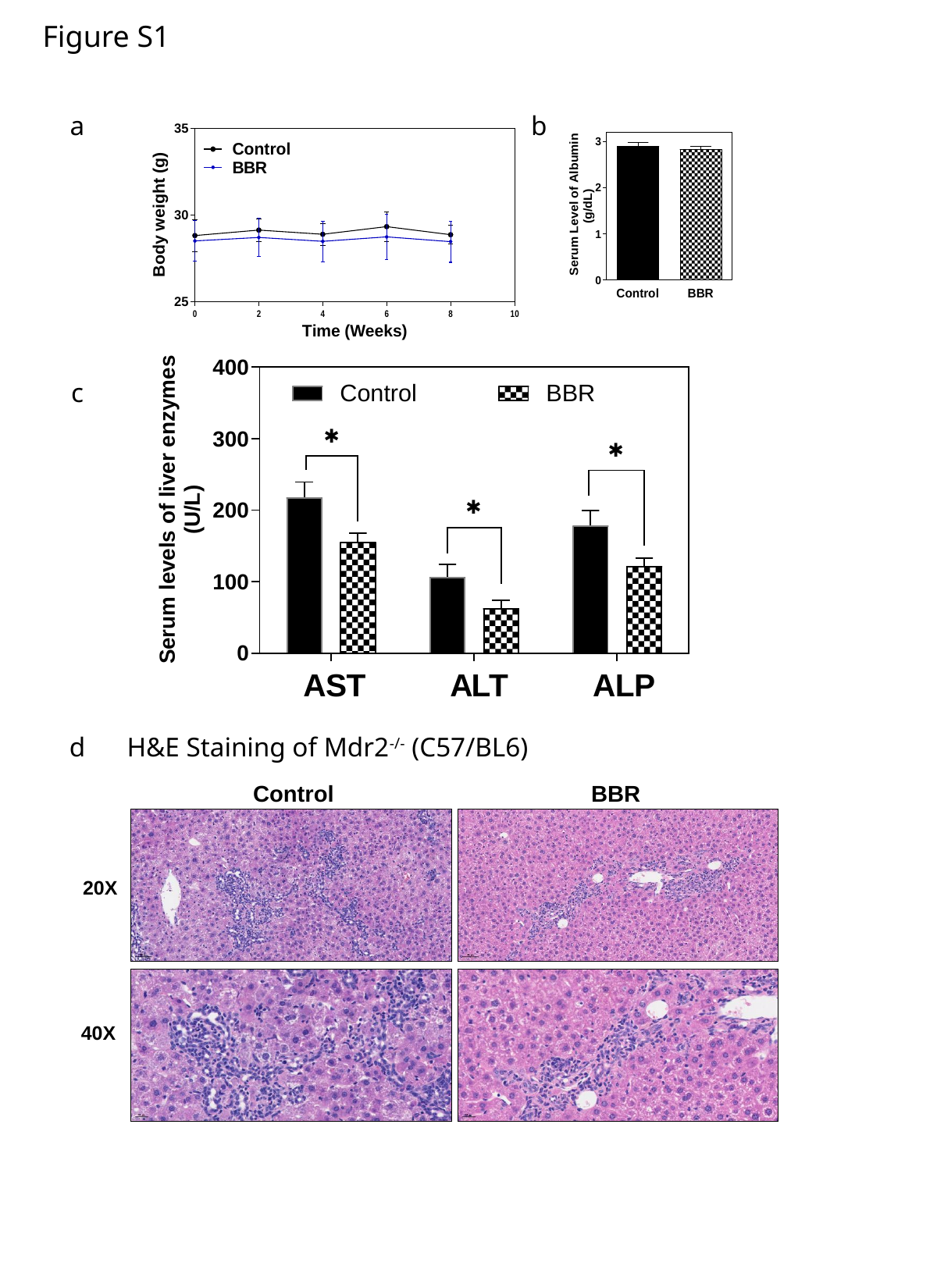

Figure S1
a
b
c
d
H&E Staining of Mdr2-/- (C57/BL6)
 Control BBR
20X
40X

## Slide 2
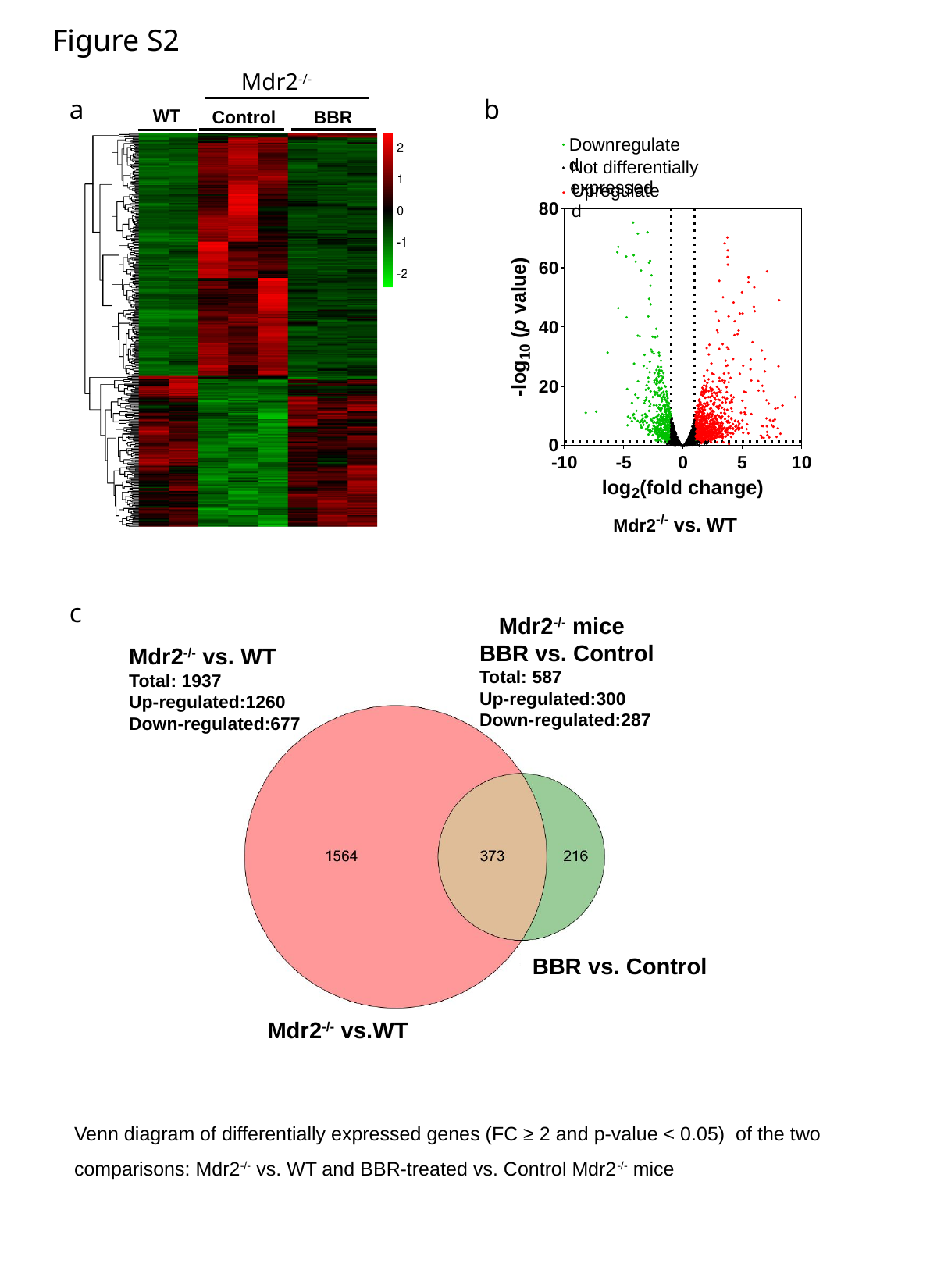

Figure S2
Mdr2-/-
a
b
 WT
 Control
 BBR
c
 Mdr2-/- mice
BBR vs. Control
Total: 587
Up-regulated:300
Down-regulated:287
Mdr2-/- vs. WT
Total: 1937
Up-regulated:1260
Down-regulated:677
BBR vs. Control
Mdr2-/- vs.WT
Venn diagram of differentially expressed genes (FC ≥ 2 and p-value < 0.05) of the two comparisons: Mdr2-/- vs. WT and BBR-treated vs. Control Mdr2-/- mice

## Slide 3
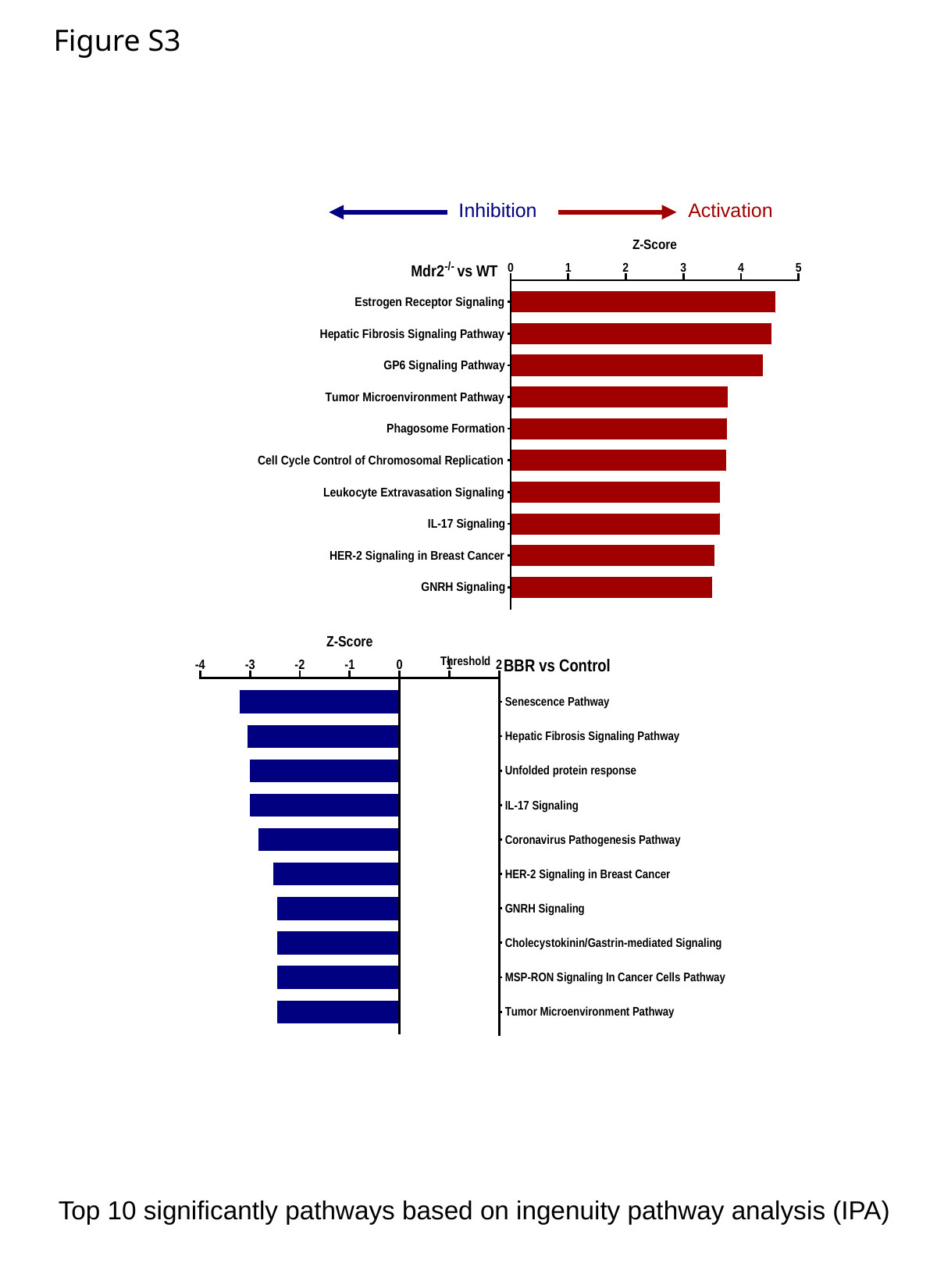

Figure S3
Inhibition
Activation
Top 10 significantly pathways based on ingenuity pathway analysis (IPA)

## Slide 4
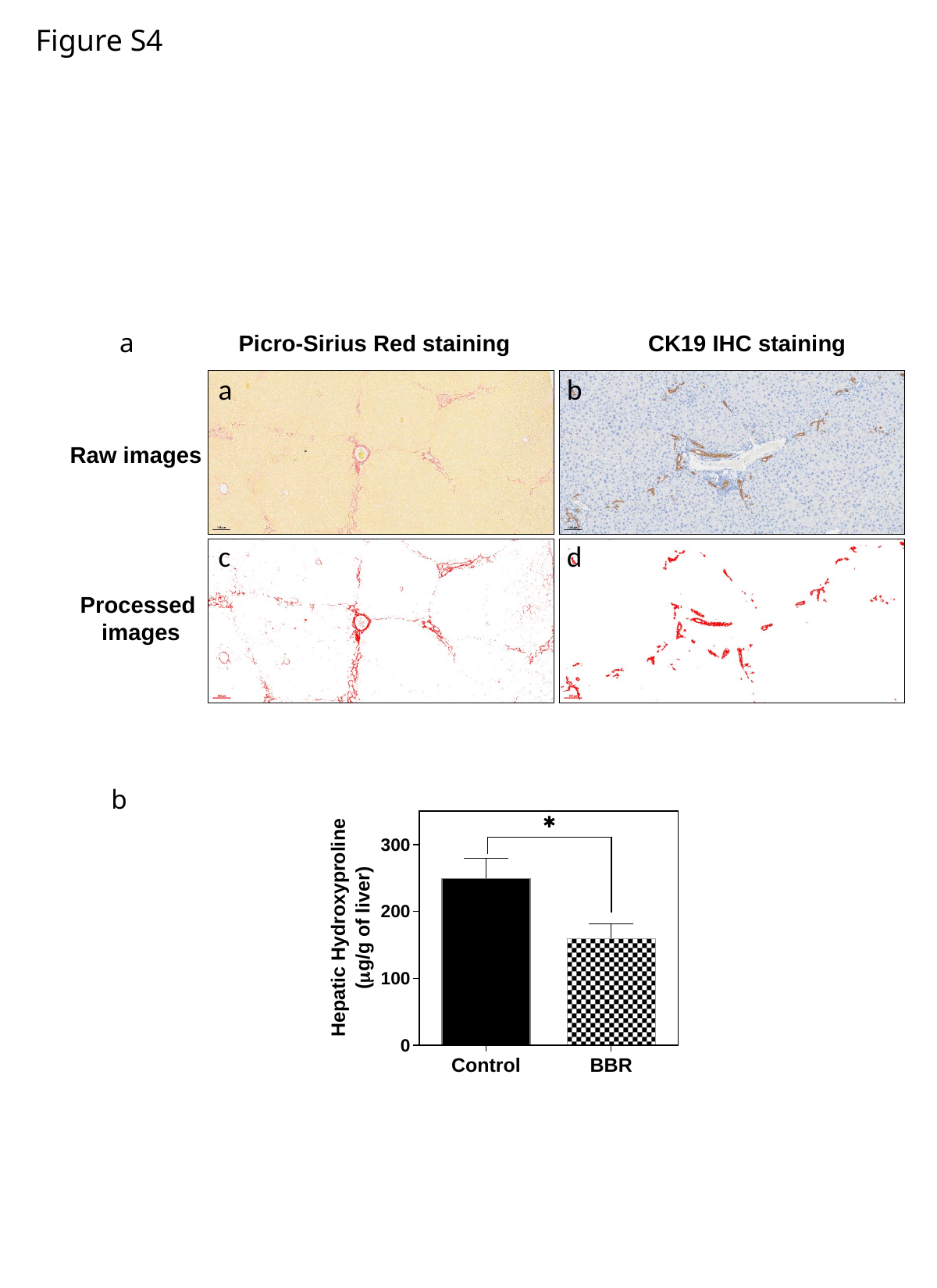

Figure S4
a
Picro-Sirius Red staining
CK19 IHC staining
a
b
Raw images
c
d
Processed
images
b

## Slide 5
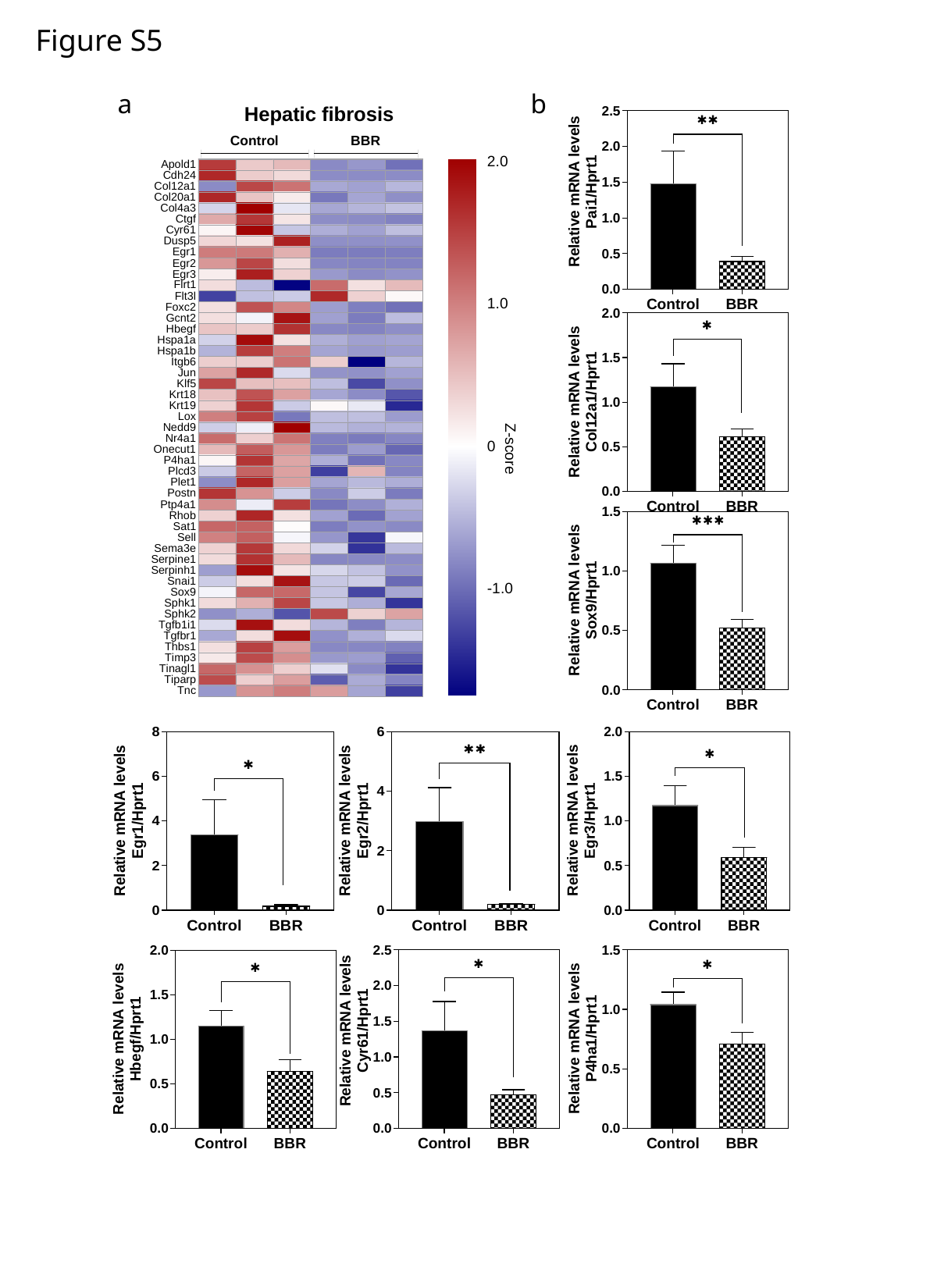

Figure S5
a
b

## Slide 6
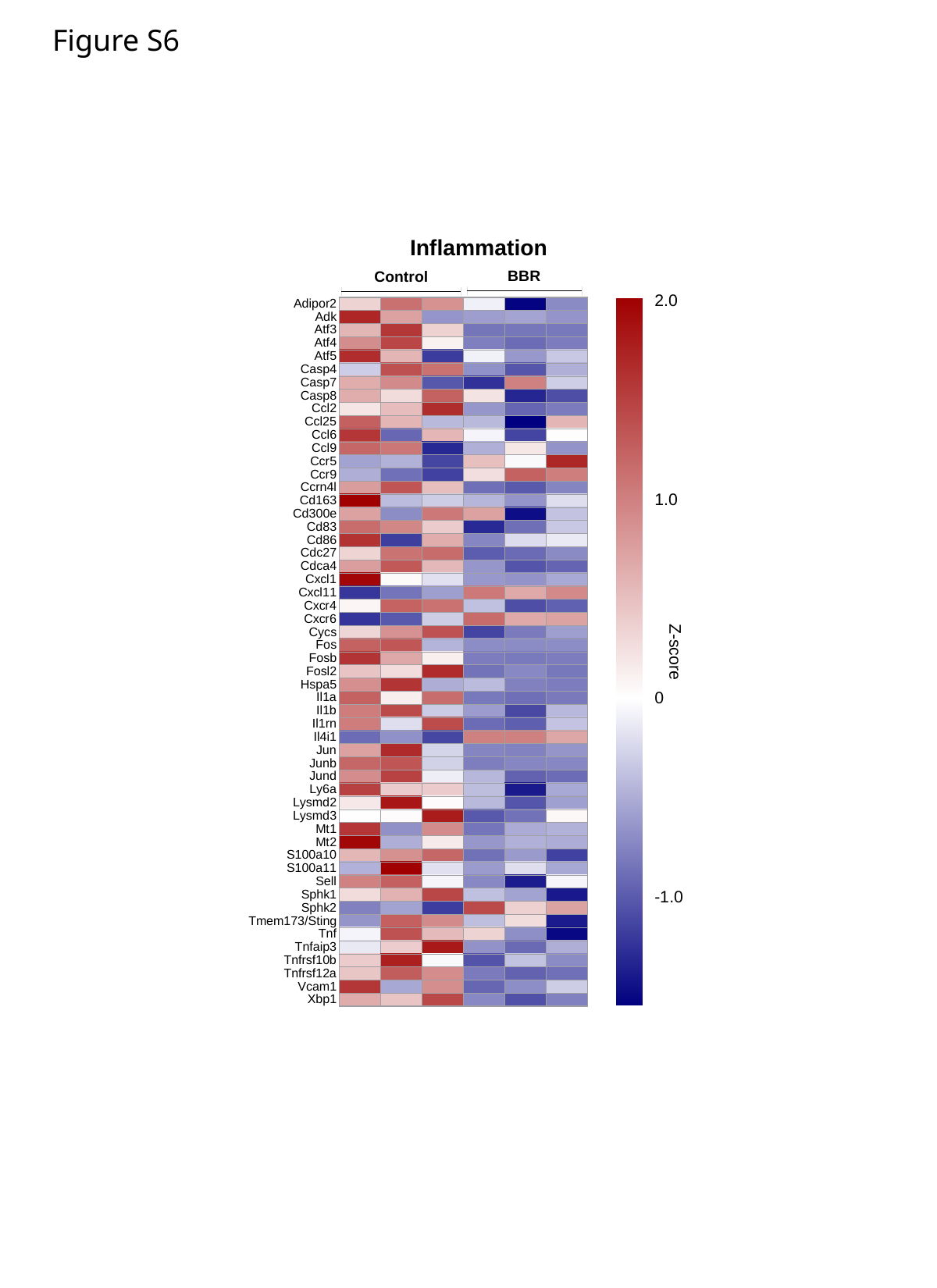

Figure S6

## Slide 7
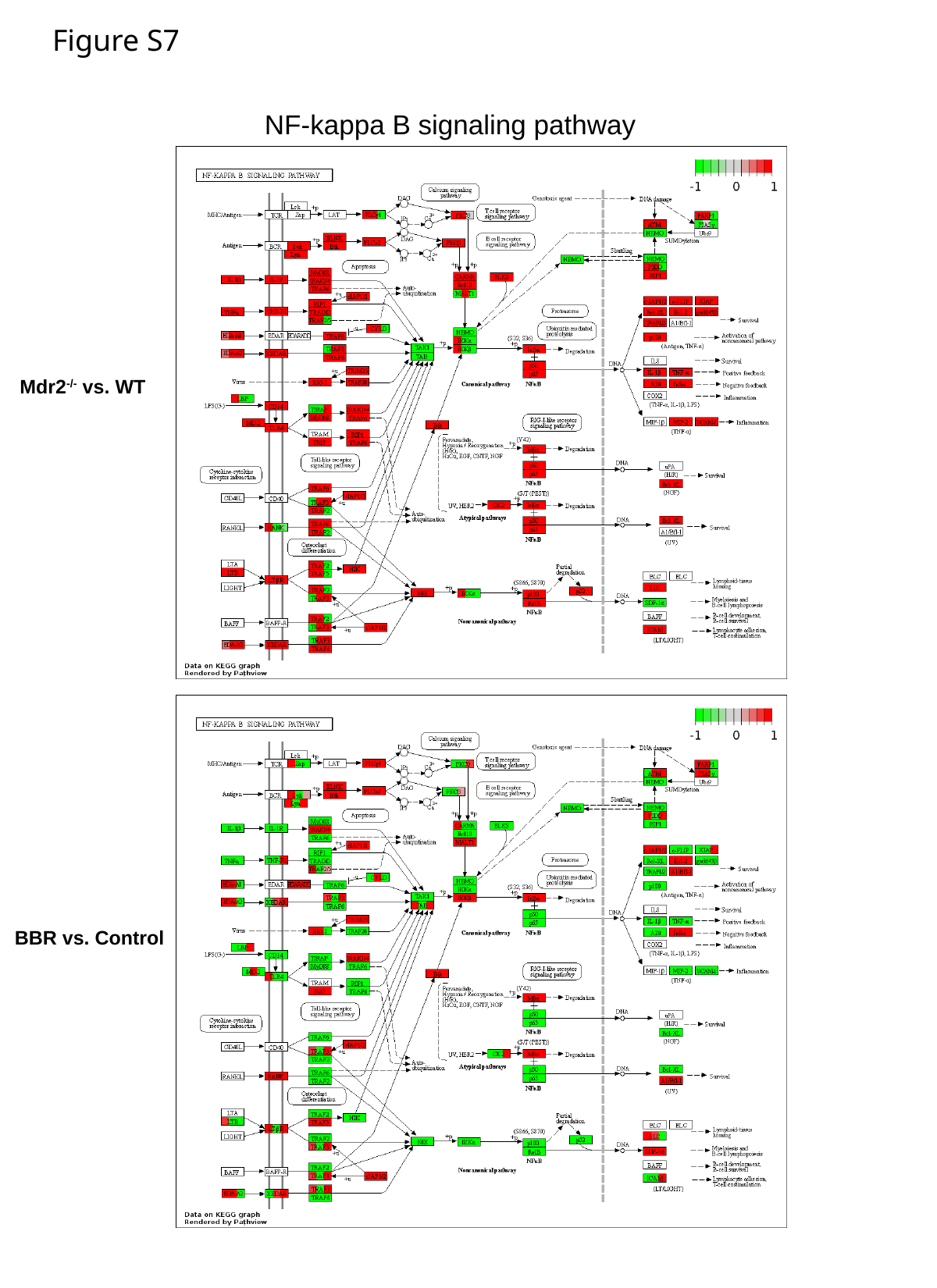

Figure S7
NF-kappa B signaling pathway
Mdr2-/- vs. WT
BBR vs. Control

## Slide 8
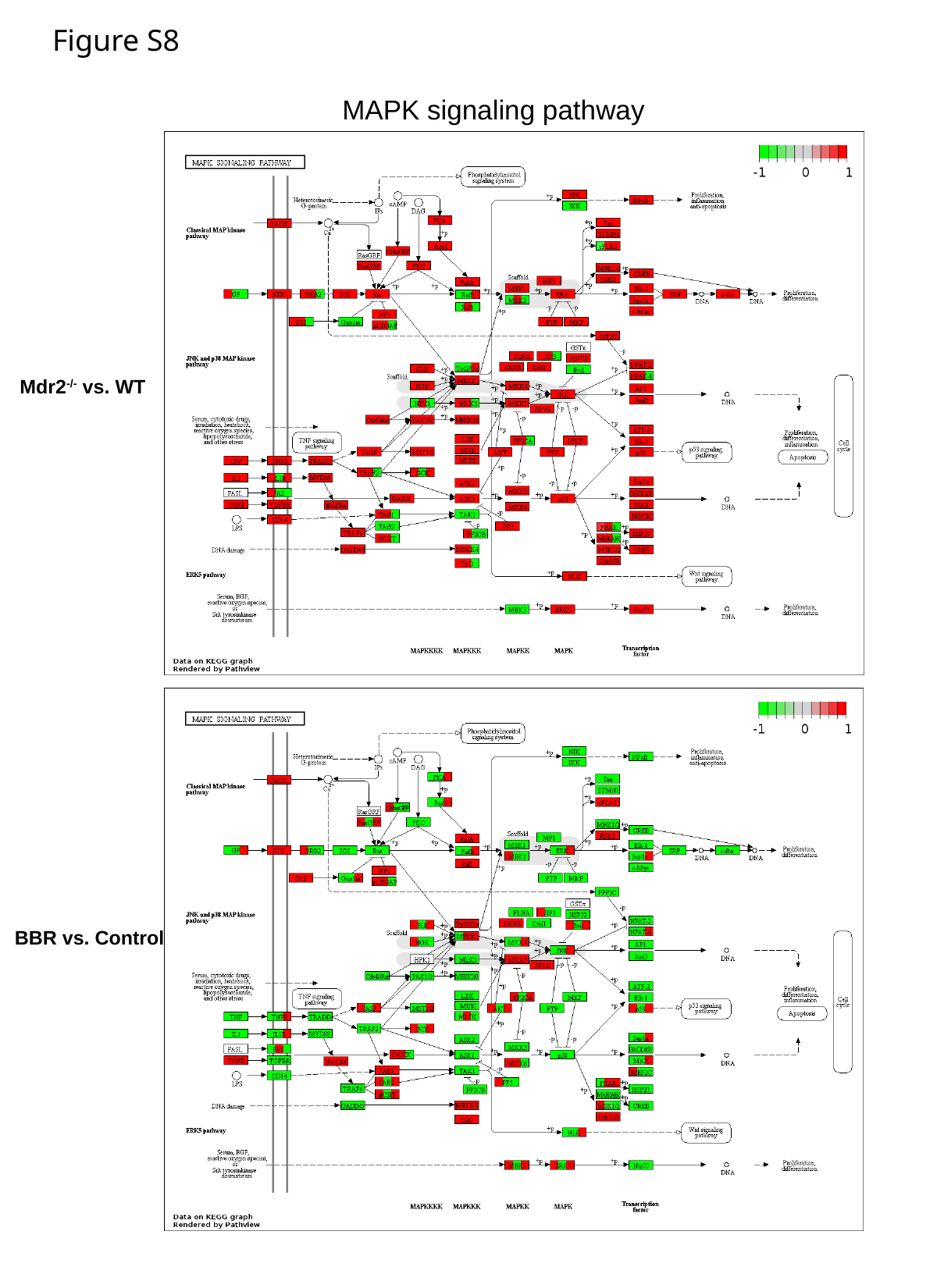

Figure S8
MAPK signaling pathway
Mdr2-/- vs. WT
BBR vs. Control

## Slide 9
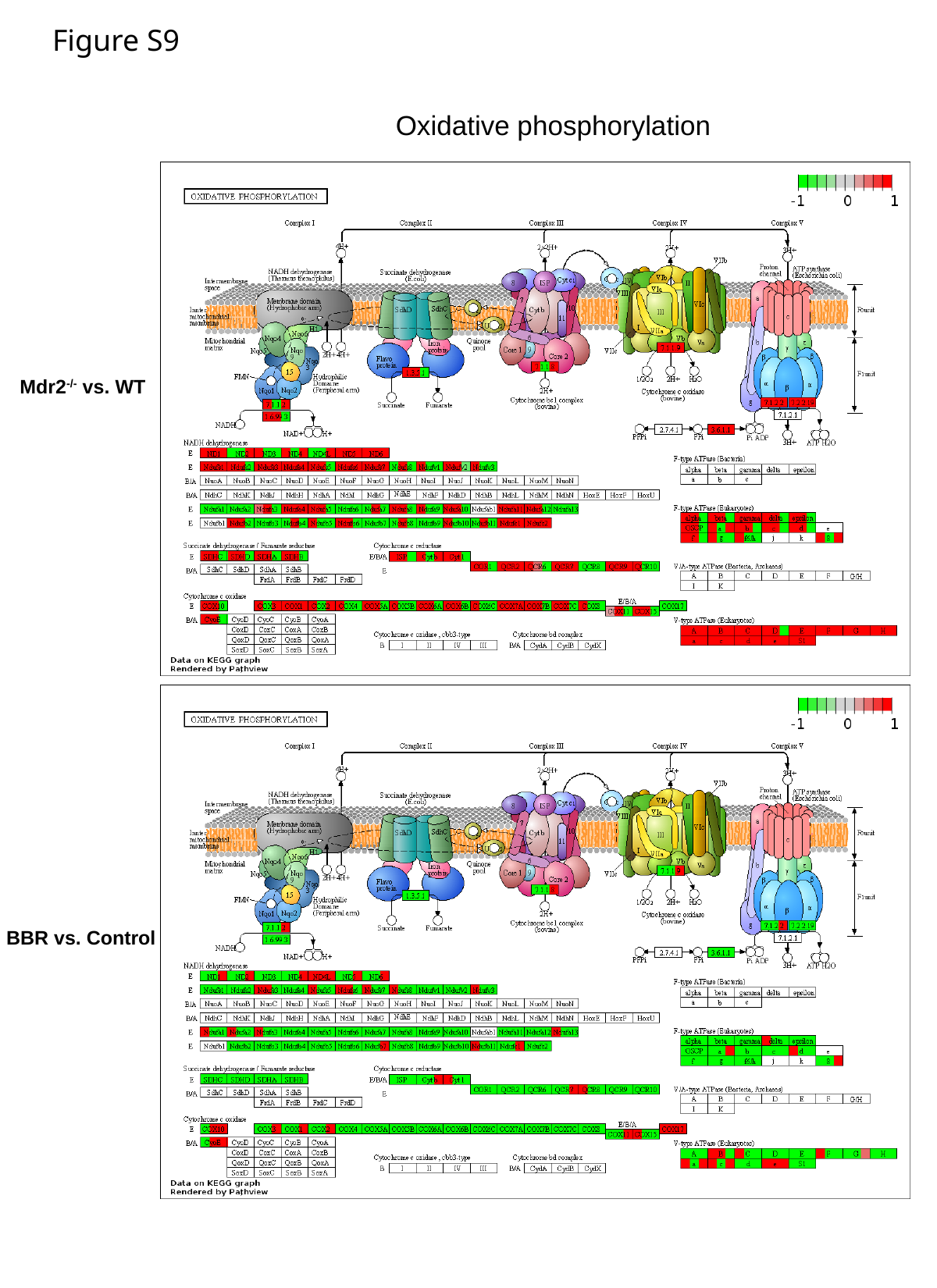

Figure S9
	Oxidative phosphorylation
Mdr2-/- vs. WT
BBR vs. Control

## Slide 10
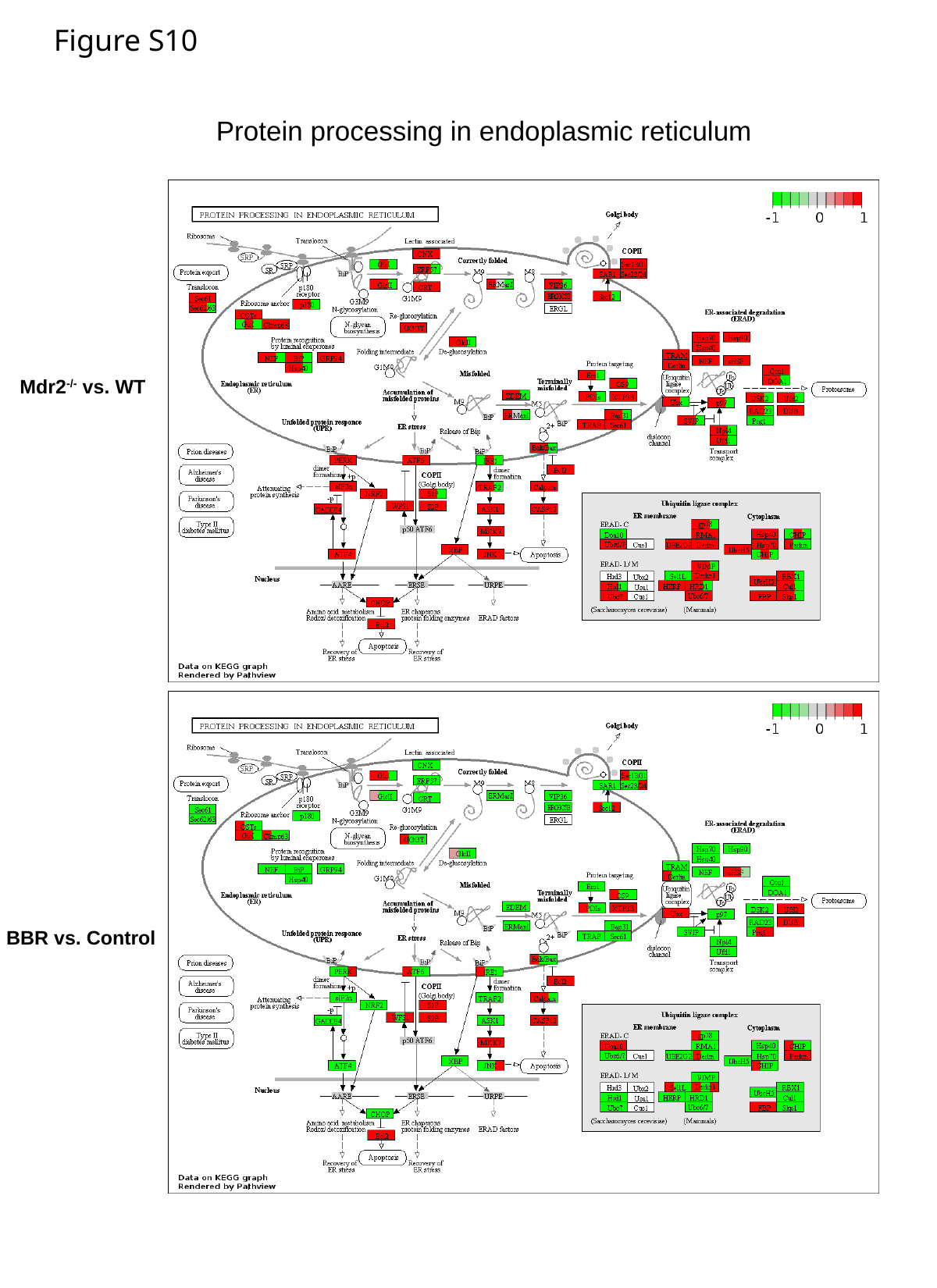

Figure S10
Protein processing in endoplasmic reticulum
Mdr2-/- vs. WT
BBR vs. Control

## Slide 11
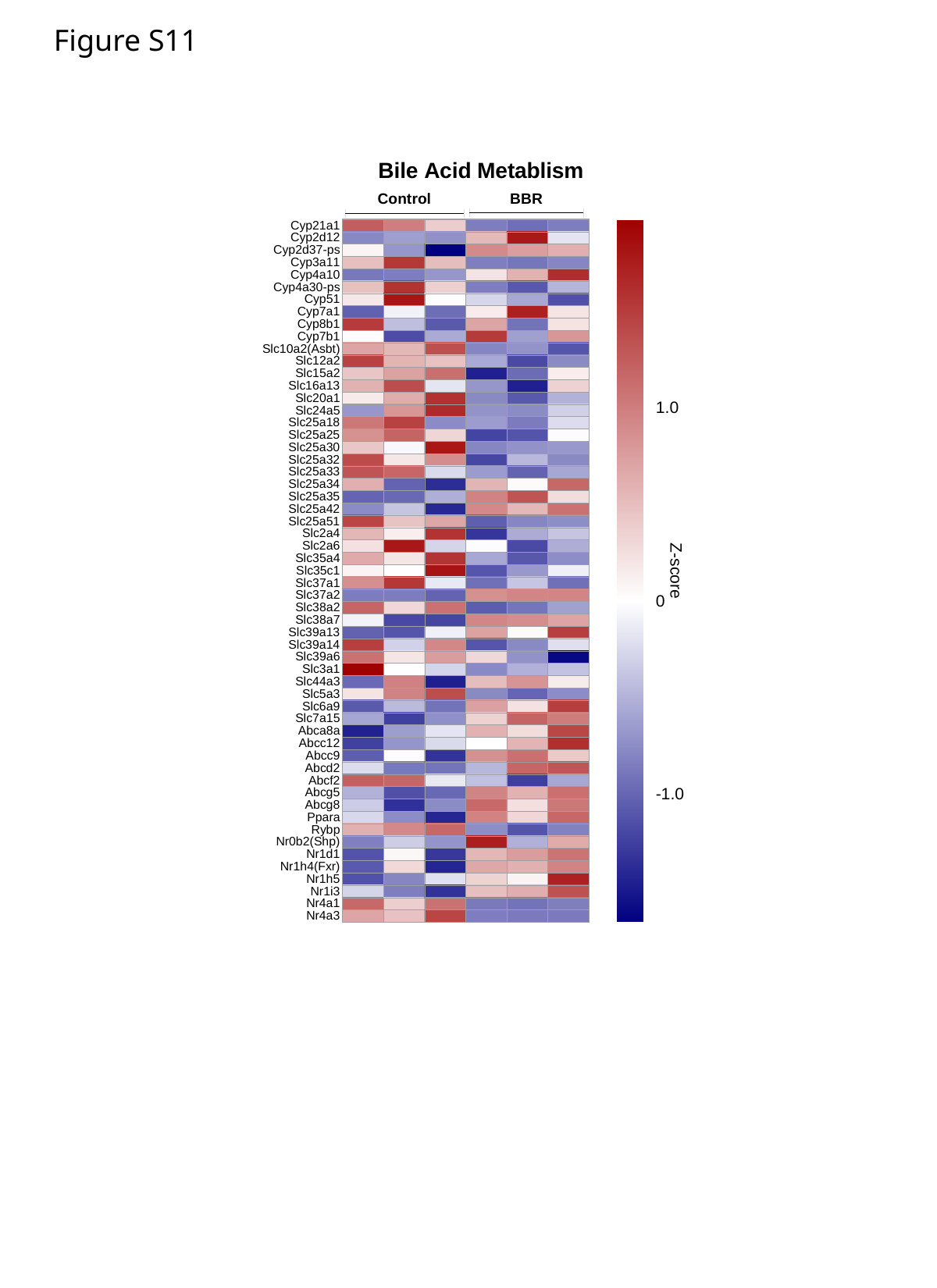

Figure S11

## Slide 12
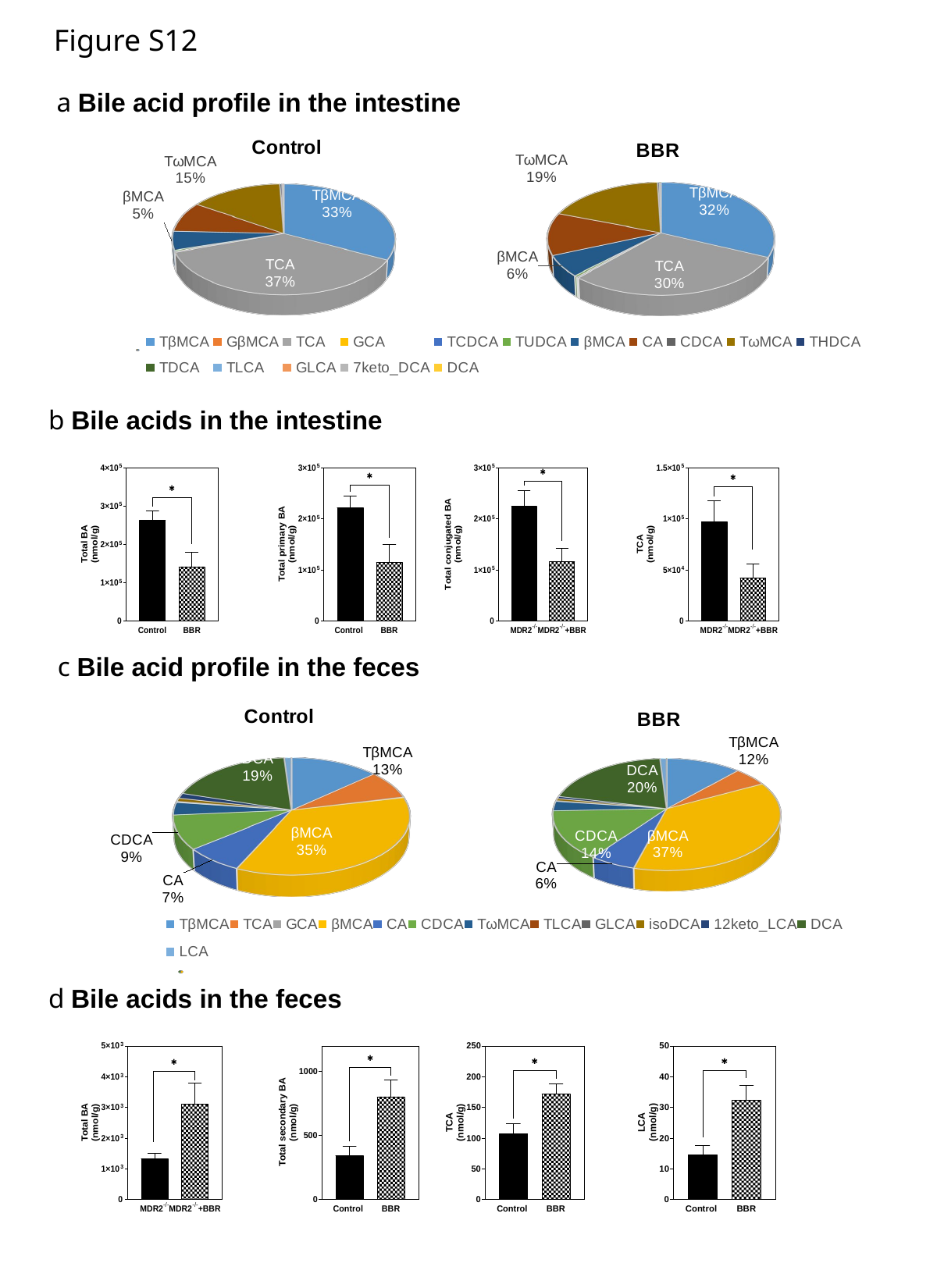

Figure S12
a Bile acid profile in the intestine
[unsupported chart]
[unsupported chart]
[unsupported chart]
b Bile acids in the intestine
c Bile acid profile in the feces
[unsupported chart]
[unsupported chart]
[unsupported chart]
d Bile acids in the feces

## Slide 13
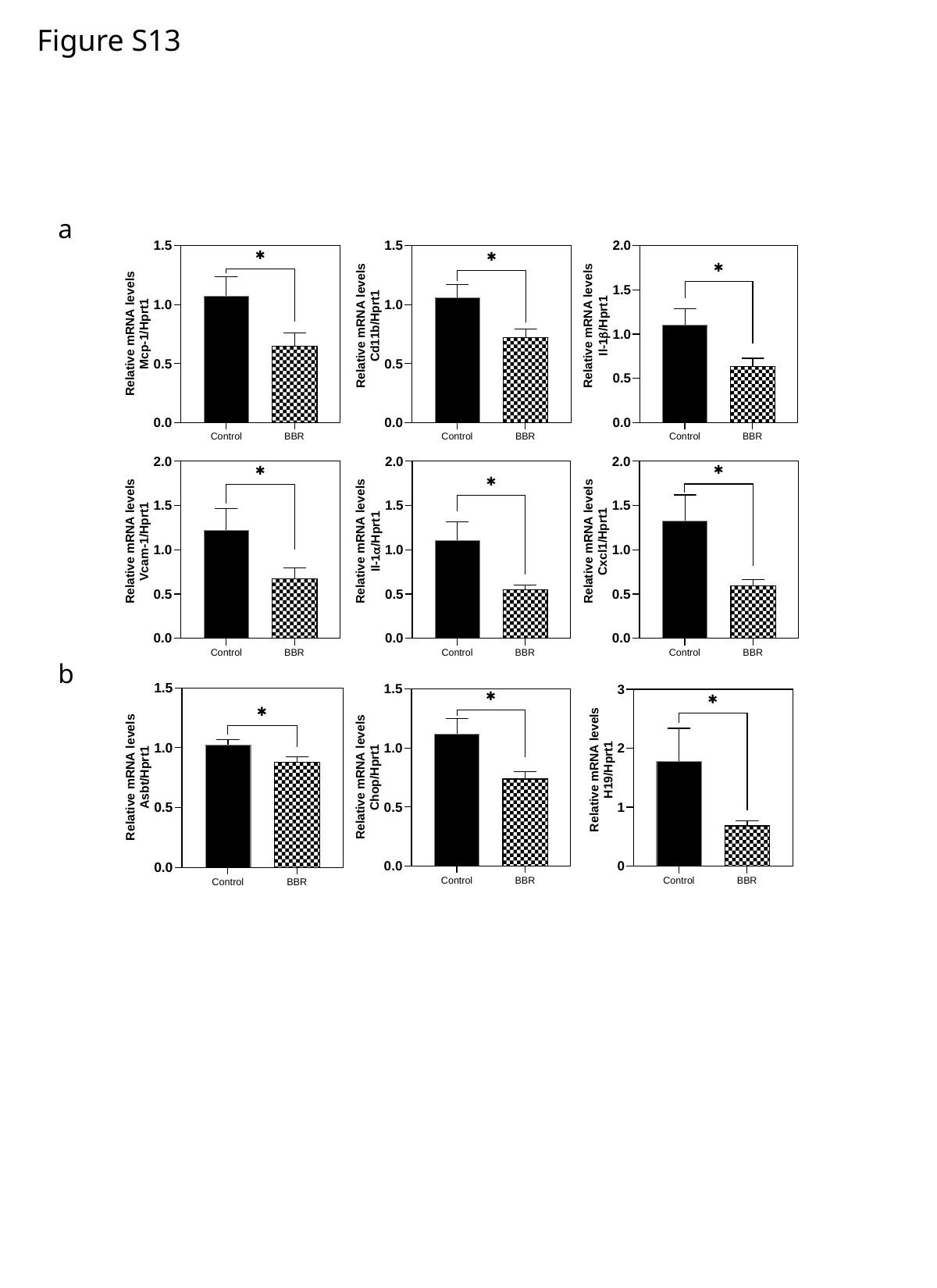

Figure S13
a
b

## Slide 14
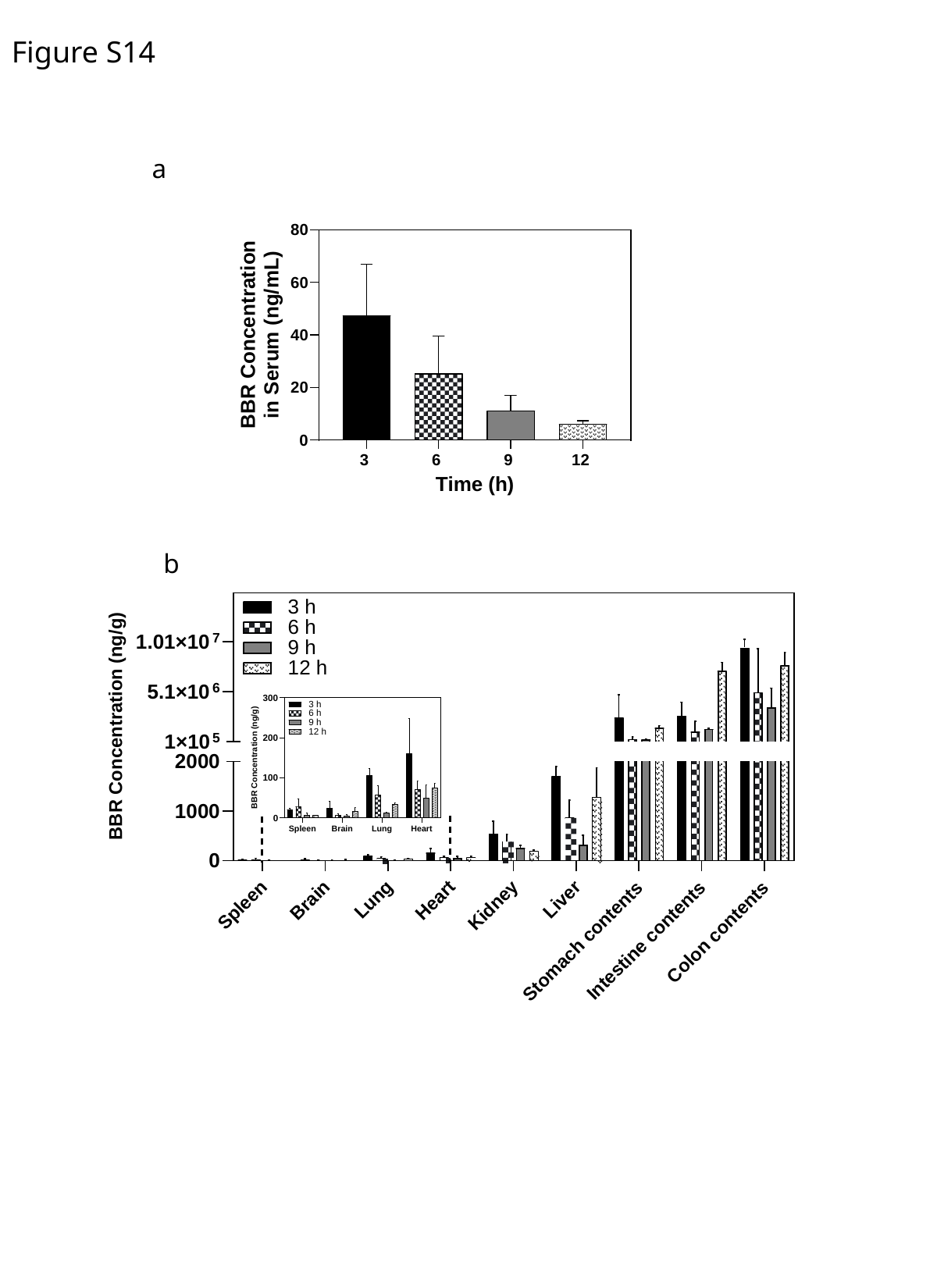

Figure S14
a
b

## Slide 15
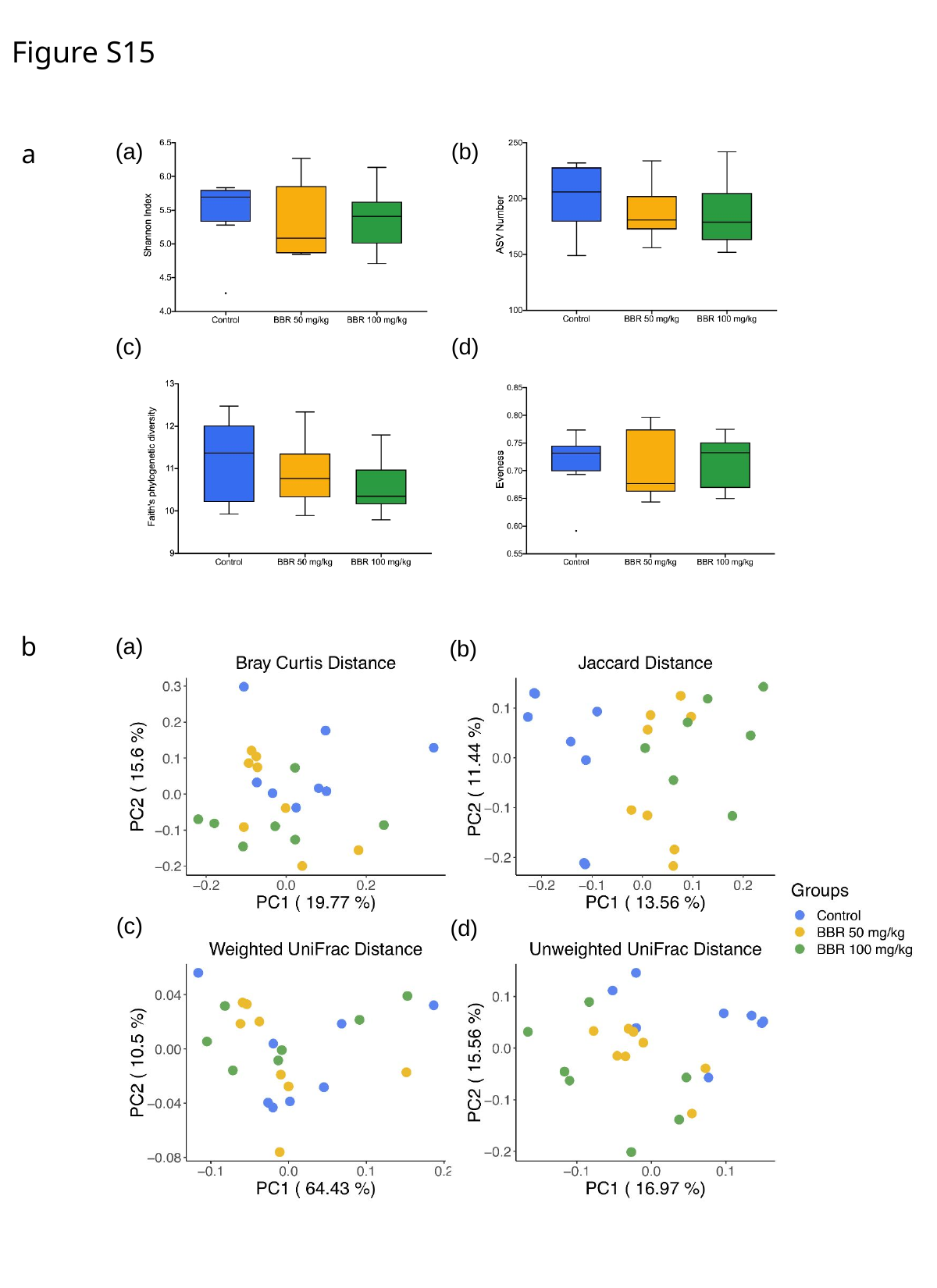

Figure S15
(a)
(b)
a
(c)
(d)
b
(a)
(b)
(c)
(d)
[unsupported chart]
[unsupported chart]

## Slide 16
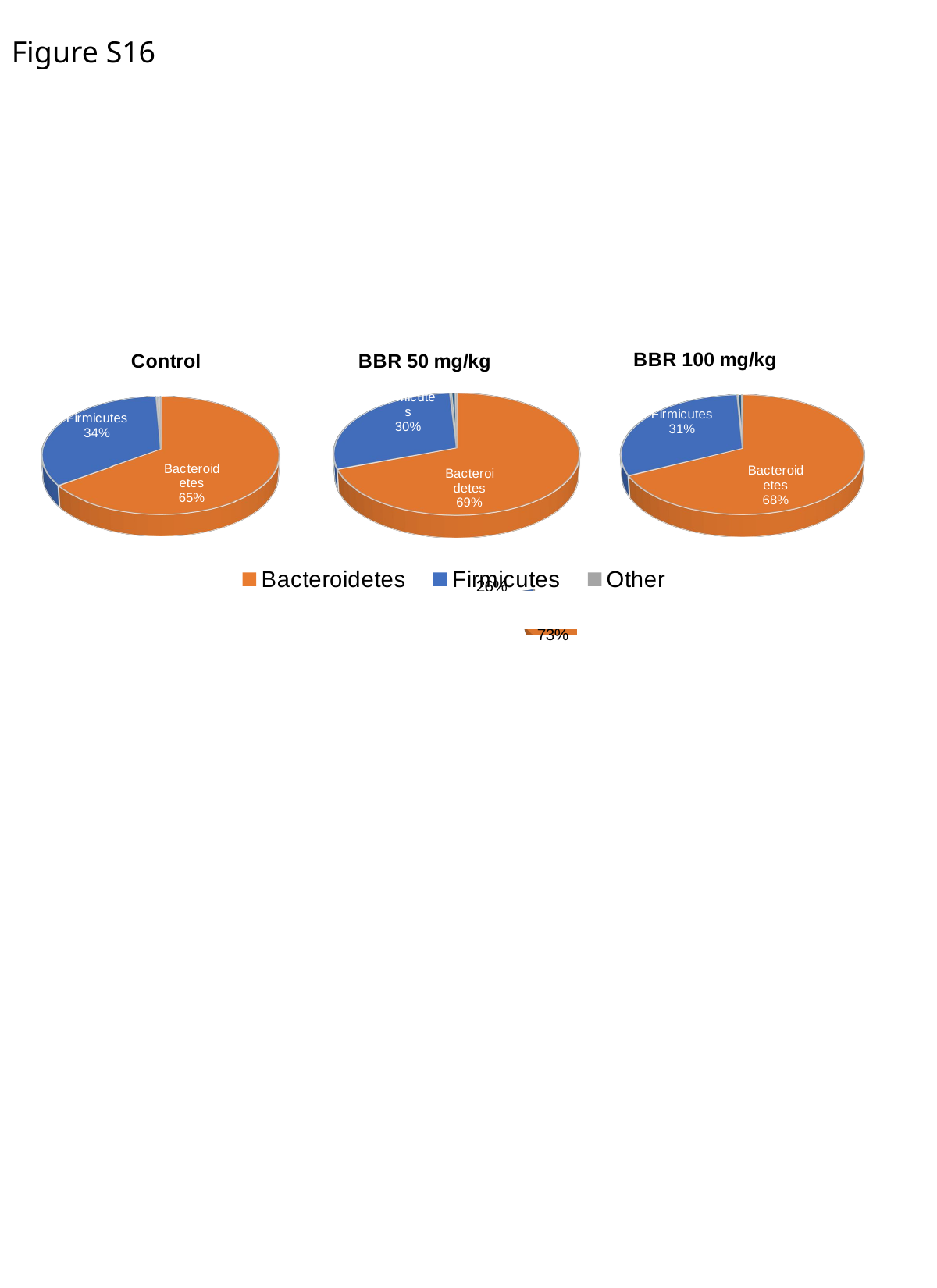

Figure S16
[unsupported chart]
[unsupported chart]
[unsupported chart]
[unsupported chart]
[unsupported chart]
[unsupported chart]
