# Supplementary material for: Therapeutic potential of berberine in attenuating cholestatic liver injury: insights from a PSC mouse model
Source: Cell Biosci. 2024 Jan 25;14:14. doi: 10.1186/s13578-024-01195-8 (PMC10809567; doi:10.1186/s13578-024-01195-8)
Supplement: Supplementary file 2 — Additional file 2. Supplement tables with captions. [file 13578_2024_1195_MOESM2_ESM.docx]

**Supplementary Tables:**

Supplementary Table S1. List of antibodies

Supplementary Table S2: Bile acid contents in the serum

Supplementary Table S3: Bile acid profile in the serum

Supplementary Table S4: Bile acid contents in the liver

Supplementary Table S5: Bile acid profile in the liver

Supplementary Table S6: Bile acid contents in the small intestine

Supplementary Table S7: Bile acids profile in the small intestine

Supplementary Table S8: Bile acid contents in the feces

Supplementary Table S9: Bile acid profile in the feces

Supplementary Table S10: BBR tissue distribution in Mdr2^−/−^mice

Supplementary Table S11: PERMANOVA test on Beta diversity

**Supplementary Table S1. List of antibodies**

| **Antibody** | **Species** | **Source** | **Catalog #** | **Application/ dilution** |
| --- | --- | --- | --- | --- |
| ATF4 | Mouse | Santa Cruz | sc-390063 | WB (1:500) |
| CHOP | Mouse | Santa Cruz | sc-7351 | WB (1:500) |
| CK19 (TROMA-III) | Rat | DSHB University of Iowa | TROMA-III | IHC (1:50) |
| CYP7A1 | Mouse | Santa Cruz | sc-518007 | WB (1:500) |
| ERK1 | Mouse | Santa Cruz | sc-271269 | WB (1:500) |
| ERK2 | Mouse | Santa Cruz | sc-1647 | WB (1:500) |
| F4/80 | Rabbit | Cell Signaling | 70076S | IHC (1:200) |
| FXR | Mouse | Santa Cruz | sc-13063 | WB (1:500) |
| Histone H3 | Rabbit | Cell Signaling | 9715S | WB (1:1000) |
| Ki67 | Rabbit | Cell Signaling | 12202S | IHC (1:200) |
| Lamin B | Mouse | Santa Cruz | sc-374015 | WB (1:500) |
| p-ERK | Mouse | Santa Cruz | sc-7383 | WB (1:500) |
| SHP | Mouse | Santa Cruz | sc-271511 | WB (1:500) |
| XBP-1 | Mouse | Santa Cruz | sc-8015 | WB (1:500) |
| β-actin (JLA20) | Mouse | DSHB University of Iowa | JLA20 | WB (1:500) |
| anti-Rabbit IgG  (H+L)-HRP | Goat | Invitrogen | 365-6120 | WB (1:2500) |
| anti-Mouse IgG(H+L)-HRP | Goat | Bio-RAD | 170-6516 | WB (1:2500) |
| anti-Rat IgG Antibody (H+L) | Rabbit | Vector Laboratories | BA-4000 | IHC (1:1000) |

Table S1: The detailed information of antibodies used in this study is listed.

| Supplementary Table S2: Bile acid contents in the serum (Mean±SD, µmol/L) | | |
| --- | --- | --- |
| **Bile Acids** | **Control** | **BBR** |
| TαMCA | 1.25±0.57 | 1.60±1.08 |
| TβMCA | 38.51±24.51 | 30.38±21.71 |
| TCA | 89.29±45.62 | 45.28±25.75* |
| GCA | 0.18±0.10 | 0.15±0.04 |
| TCDCA | 0.40±0.20 | 0.40±0.20 |
| GβMCA | 0.10±0.00 | 0.10±0.01 |
| βMCA | 1.55±1.21 | 1.62±0.89 |
| CA | 0.77±0.80 | 1.09±1.22 |
| CDCA | 0.10±0.01 | 0.10±0.01 |
| TωMCA | 20.97±15.88 | 10.49±5.73* |
| TDCA | 0.40±0.25 | 0.48±0.34 |
| TLCA | 0.05±0.01 | 0.05±0.00 |
| GLCA | 0.12±0.00 | 0.12±0.01 |
| TUDCA | 0.43±0.49 | 0.37±0.19 |
| THDCA | 0.72±0.95 | 0.33±0.28 |
| ωMCA | 0.85±0.80 | 0.99±0.51 |
| DCA | 0.32±0.30 | 0.26±0.15 |
| LCA | 0.13±0.00 | 0.13±0.00 |

Table S2: The serum levels of individual BA were measured by LC-MS/MS. The statistical significance between Mdr2^-/-^ control group and Mdr2^-/-^treated with BBR was analyzed using the paired Student t-test, *p<0.05 relative to the control group.

| Supplementary Table S3: Bile acid profile in the serum (Mean±SD, nmol/L) | | |
| --- | --- | --- |
| **Bile Acids** | **Control** | **BBR** |
| Total BA | 155.85±76.71 | 93.90±51.20* |
| Total primary BA | 131.94±64.44 | 80.69±46.18* |
| Total primary conjugated BA | 129.56±63.19 | 77.91±45.00* |
| Total primary unconjugated BA | 2.37±1.78 | 2.78±2.08 |
| Total secondary BA | 23.91±17.67 | 13.21±6.61 |
| Total secondary conjugated BA | 25.27±16.01 | 11.84±6.10 |
| Total secondary unconjugated BA | 1.36±0.86 | 1.37±0.64 |
| Total conjugated BA | 152.22±74.97 | 89.75±49.71* |
| Total unconjugated BA | 3.63±2.36 | 4.15±2.41 |
| Ratio of total primary BA to total BA | 0.85±0.07 | 0.84±0.06 |
| Ratio of total primary BA to total secondary BA | 5.54±3.02 | 6.03±2.99 |
| Ratio of total primary conjugated BA to total primary unconjugated BA | 73.65±46.32 | 32.91±18.97* |
| Ratio of total conjugated BA to total unconjugated BA | 49.55±22.96 | 23.02±11.89** |
| Ratio of total secondary BA to total BA | 0.17±0.05 | 0.16±0.06 |
| Ratio of total secondary conjugated BA to total secondary unconjugated BA | 19.14±7.05 | 9.55±5.27* |

Table S3. The BA profile in the serum was analyzed, including total BA, primary BA, secondary BA, conjugated and unconjugated, as well as the ratio of different BA. The statistical significance between Mdr2^-/-^ control group and Mdr2^-/-^treated with BBR was analyzed using the paired Student t-test, *p<0.05, **p<0.01, relative to the control group.

| Supplementary Table S4: Bile acid contents in the liver (Mean±SD, nmol/g) | | |
| --- | --- | --- |
| **Bile Acids** | **Control** | **BBR** |
| TβMCA | 446.80±103.74 | 368.57±97.10 |
| GβMCA | 0.17±0.05 | 0.15±0.08 |
| TCA | 593.00±196.81 | 379.20±97.38** |
| GCA | 0.24±0.24 | 0.17±0.14 |
| TCDCA | 3.27±1.53 | 3.52±1.65 |
| CA | 0.90±1.04 | 0.35±0.23 |
| αMCA | 0.64±0.72 | 0.09±0.06* |
| βMCA | 25.86±20.08 | 12.38±6.72 |
| TωMCA | 207.54±77.59 | 149.17±115.05 |
| TDCA | 4.13±1.79 | 1.79±2.05* |
| TUDCA | 4.34±1.62 | 4.21±2.28 |
| THDCA | 4.79±3.51 | 2.65±1.93 |
| GDCA | 0.03±0.01 | 0.03±0.01 |
| TLCA | 0.05±0.02 | 0.03±0.02** |
| 7keto_DCA | 0.04±0.03 | 0.02±0.02 |
| ωMCA | 5.69±6.61 | 1.87±1.73 |
| DCA | 0.62±0.43 | 0.42±0.16 |

Table S4: The levels of individual BA in the liver were measured by LC-MS/MS. The statistical significance between Mdr2^-/-^ control group and Mdr2^-/-^treated with BBR was analyzed using the paired Student t-test, *p<0.05, **p<0.01, relative to the control group.

Table S5. The BA profile in the liver was analyzed, including total BA, primary BA, secondary BA, conjugated and unconjugated, as well as the ratio of different BA. The statistical significance between Mdr2^-/-^ control group and Mdr2^-/-^treated with BBR was analyzed using the paired Student t-test, *p<0.05, relative to the control group.

| Supplementary Table S5: Bile acid profile in the liver (Mean±SD, nmol/g) | | |
| --- | --- | --- |
| **Bile Acids** | **Control** | **BBR** |
| Total BA | 1,200.93±193.48 | 924.60±233.52* |
| Total primary BA | 999.56±198.35 | 764.42±184.81* |
| Total primary conjugated BA | 979.14±199.95 | 751.61±183.00* |
| Total primary unconjugated BA | 20.42±7.69 | 12.81±6.61 |
| Total secondary BA | 201.37±32.09 | 160.18±119.76 |
| Total secondary conjugated BA | 197.31±31.12 | 157.89±118.07 |
| Total secondary unconjugated BA | 4.06±2.12 | 2.29±1.77 |
| Total conjugated BA | 1,176.45±196.00 | 909.50±228.32* |
| Total unconjugated BA | 24.48±9.57 | 15.10±8.27 |
| Ratio of total primary BA to total BA | 0.83±0.04 | 0.83±0.09 |
| Ratio of total primary BA to total secondary BA | 5.10±1.36 | 7.94±6.95 |
| Ratio of total primary conjugated BA to total primary unconjugated BA | 56.88±28.67 | 67.88±24.98 |
| Ratio of total conjugated BA to total unconjugated BA | 57.20±28.37 | 69.04±22.46 |
| Ratio of total secondary BA to total BA | 0.17±0.04 | 0.17±0.09 |
| Ratio of total secondary conjugated BA to total secondary unconjugated BA | 58.84±27.19 | 69.73±14.66 |

| Supplementary Table S6: Bile acid contents in the small intestine (Mean±SD, nmol/g) | | |
| --- | --- | --- |
| **Bile Acids** | **Control** | **BBR** |
| TβMCA | 85,755.72±23,880.74 | 45,609.48±44,619.10* |
| GβMCA | 40.18±22.67 | 30.27±27.20 |
| TCA | 97,557.79±57,475.83 | 42,515.86±35,709.13* |
| GCA | 83.05±76.84 | 71.10±110.13 |
| TCDCA | 581.55±449.37 | 345.71±384.14 |
| TUDCA | 803.37±553.18 | 575.99±388.61 |
| βMCA | 14,361.81±11,793.75 | 8,812.81±14,996.41 |
| CA | 23,154.28±28,375.80 | 17,371.91±32,192.63 |
| CDCA | 39.55±36.26 | 22.80±14.94 |
| TωMCA | 39,319.10±18,453.02 | 26,765.19±22,808.64 |
| THDCA | 853.94±220.99 | 415.06±332.05** |
| TDCA | 541.18±542.79 | 240.56±316.70 |
| TLCA | 5.62±1.16 | 5.82±1.77 |
| GLCA | 5.58±3.70 | 8.72±1.56 |
| 7keto_DCA | 72.95±37.69 | 45.75±23.02 |
| DCA | 62.26±67.63 | 24.43±33.17 |

Table S6: The levels of individual BA in the small intestine were measured by LC-MS/MS. The statistical significance between Mdr2^-/-^ control group and Mdr2^-/-^treated with BBR was analyzed using the paired Student t-test, *p<0.05, **p<0.01, relative to the control group.

Table S7. The BA profile in the small intestine was analyzed, including total BA, primary BA, secondary BA, conjugated and unconjugated, as well as the ratio of different BA. The statistical significance between Mdr2^-/-^ control group and Mdr2^-/-^treated with BBR was analyzed using the paired Student t-test, *p<0.05, relative to the control group.

| Supplementary Table S7: Bile acid profile in the small intestine (Mean±SD, nmol/g) | | |
| --- | --- | --- |
| **Bile Acids** | **Control** | **BBR** |
| Total BA | 263,233.74±70,684.66 | 142,750.71±96,152.20* |
| Total primary BA | 222,377.29±61,467.47 | 115,252.65±92,976.30* |
| Total primary conjugated BA | 184,821.65±67,474.30 | 89,051.65±58,696.45* |
| Total primary unconjugated BA | 37,555.64±39,928.42 | 26,201.00±47,156.55 |
| Total secondary BA | 40,856.45±18,749.38 | 27,498.06±23,219.06 |
| Total secondary conjugated BA | 40,794.18±18,748.03 | 27,473.63±23,208.18 |
| Total secondary unconjugated BA | 62.26±67.63 | 24.43±33.17 |
| Total conjugated BA | 225,615.83±82,741.69 | 116,525.28±68,153.78* |
| Total unconjugated BA | 37,617.91±39,945.25 | 26,225.43±47,157.49 |
| Ratio of total primary BA to total BA | 0.84±0.06 | 0.77±0.13 |
| Ratio of total primary BA to total secondary BA | 8.56±10.49 | 7.86±12.38 |
| Ratio of total primary conjugated BA to total primary unconjugated BA | 26.79±39.63 | 15.01±12.39 |
| Ratio of total conjugated BA to total unconjugated BA | 32.56±47.81 | 20.46±15.75 |
| Ratio of total secondary BA to total BA | 0.16±0.06 | 0.23±0.13 |
| Ratio of total secondary conjugated BA to total secondary unconjugated BA | 2,038.94±1,917.41 | 2,282.91±3,375.76 |

| Supplementary Table S8: Bile acid contents in the feces (Mean±SD, nmol/g) | | |
| --- | --- | --- |
| **Bile Acids** | **Control** | **BBR** |
| TβMCA | 183.70±69.29 | 373.47±463.92 |
| TCA | 107.67±42.46 | 172.00±46.37* |
| GCA | 2.20±0.09 | 1.81±0.11 |
| βMCA | 485.47±300.82 | 1,172.37±1,389.87 |
| CA | 100.00±119.38 | 186.89±139.64 |
| CDCA | 129.43±90.74 | 452.74±222.99** |
| TωMCA | 48.01±20.65 | 86.65±46.41* |
| TLCA | 2.01±0.45 | 2.14±0.38 |
| GLCA | 1.89 | 4.14±2.07 |
| isoDCA | 12.58±3.15 | 21.42±10.87 |
| 12keto_LCA | 19.74±17.89 | 20.80±24.92 |
| DCA | 261.94±149.80 | 644.69±328.99* |
| LCA | 14.69±13.14 | 32.41±12.60* |

Table S8: The levels of individual BA in the feces were measured by LC-MS/MS. The statistical significance between Mdr2^-/-^ control group and Mdr2^-/-^treated with BBR was analyzed using the paired Student t-test, *p<0.05, **p<0.01, relative to the control group.

Table S9. The BA profile in the feces was analyzed, including total BA, primary BA, secondary BA, conjugated and unconjugated, as well as the ratio of different BA. The statistical significance between Mdr2^-/-^ control group and Mdr2^-/-^treated with BBR was analyzed using the paired Student t-test, *p<0.05, **p<0.01, relative to the control group.

| Supplementary Table S9: Bile acid profile in the feces (Mean±SD, nmol/g) | | |
| --- | --- | --- |
| **Bile Acids** | **Control** | **BBR** |
| Total BA | 1,332.76±472.17 | 3,107.18±1,967.15* |
| Total primary BA | 988.72±452.21 | 2,301.33±1,686.65* |
| Total primary conjugated BA | 292.31±80.96 | 545.92±482.81 |
| Total primary unconjugated BA | 696.41±415.18 | 1,755.40±1,633.91 |
| Total secondary BA | 344.03±188.74 | 805.85±360.34** |
| Total secondary conjugated BA | 69.50±34.19 | 132.80±51.59* |
| Total secondary unconjugated BA | 274.54±162.53 | 673.05±341.41* |
| Total conjugated BA | 361.81±79.17 | 678.72±508.88 |
| Total unconjugated BA | 970.94±439.49 | 2,428.46±1,862.43 |
| Ratio of total primary BA to total BA | 0.73±0.11 | 0.68±0.10 |
| Ratio of total primary BA to total secondary BA | 4.46±5.33 | 2.52±1.71 |
| Ratio of total primary conjugated BA to total primary unconjugated BA | 0.57±0.42 | 0.63±0.59 |
| Ratio of total conjugated BA to total unconjugated BA | 0.45±0.26 | 0.44±0.31 |
| Ratio of total secondary BA to total BA | 0.27±0.11 | 0.32±0.10 |
| Ratio of total secondary conjugated BA to total secondary unconjugated BA | 0.28±0.12 | 0.23±0.10 |

Table S10: The tissue distribution of BBR in Mdr2^-/-^ mice. BBR (50 mg/kg) was given via oral gavage. After 3, 6, and 9 h, serum and different tissues were harvested and processed for LC-MS/MS analysis for BBR levels.

| Supplementary Table S10: BBR tissue distribution in Mdr2^−/−^mice (Mean±SD, ng/mL or ng/g) | | | |
| --- | --- | --- | --- |
| **Specimen** | **3 h** | **6 h** | **9 h** |
| Serum | 47.57±33.57 | 25.40±24.55 | 11.31±9.80 |
| Spleen | 21.60±4.95 | 29.75±25.24 | 8.30±9.36 |
| Brain | 24.35±25.53 | 7.35±5.73 | 6.43±4.94 |
| Lung | 106.00±25.46 | 58.50±31.82 | 14.00±1.00 |
| Heart | 160.60±123.60 | 71.25±30.05 | 51.23±53.53 |
| Kidney | 550.00±350.72 | 395.50±194.45 | 252.67±87.49 |
| Liver | 1,711.37±333.38 | 874.63±606.38 | 327.60±320.52 |
| Stomach contents | 2,511,320.67±3,940,463.36 | 348,617.67±409,223.83 | 336,693.33±82,463.75 |
| Intestine contents | 2,672,096.67±2,376,140.29 | 1,107,885.00±1,475,144.95 | 1,384,960.00±74,557.34 |
| Colon feces | 9,534,673.20±1,398,092.64 | 5,045,373.74±7,543,827.40 | 3,517,386.67±3,347,087.97 |

| Supplementary Table S11: PERMANOVA test on Beta diversity | | | |
| --- | --- | --- | --- |
| **Pairs** | **F. Model** | **R2** | **P.value** |
| **Bray-Curtis distance** | | | |
| Control vs. BBR 50 mg/kg | 1.659 | 0.105 | 0.039 |
| Control vs. BBR 100 mg/kg | 2.241 | 0.147 | 0.005 |
| BBR 50 mg/kg vs. BBR 100 mg/kg | 0.845 | 0.061 | 0.615 |
| **Jaccard distance** | | | |
| Control vs. BBR 50 mg/kg | 1.923 | 0.1208 | 0.005 |
| Control vs. BBR 100 mg/kg | 2.607 | 0.1671 | 0.001 |
| BBR 50 mg/kg vs. BBR 100 mg/kg | 1.038 | 0.0739 | 0.391 |
| **Weighted UniFrac distance** | | | |
| Control vs. BBR 50 mg/kg | 0.8229 | 0.0555 | 0.442 |
| Control vs. BBR 100 mg/kg | 1.0277 | 0.0732 | 0.345 |
| BBR 50 mg/kg vs. BBR 100 mg/kg | 0.244 | 0.0184 | 0.919 |
| **Unweighted UniFrac distance** | | | |
| Control vs. BBR 50 mg/kg | 1.858 | 0.1172 | 0.019 |
| Control vs. BBR 100 mg/kg | 2.899 | 0.1823 | 0.002 |
| BBR 50 mg/kg vs. BBR 100 mg/kg | 1.142 | 0.0807 | 0.299 |

Table S11: The Beta diversity of gut microbiome was analyzed using PERMANOVA.
